# Cryo–light microscopy with angstrom precision deciphers structural conformations of PIEZO1 in its native state

**DOI:** 10.1126/sciadv.adw4402

**Published:** 2025-08-20

**Authors:** Hisham Mazal, Franz-Ferdinand Wieser, Daniel Bollschweiler, Alexandra Schambony, Vahid Sandoghdar

**Affiliations:** ^1^ Max Planck Institute for the Science of Light, 91058 Erlangen, Germany.; ^2^ Max-Planck-Zentrum für Physik und Medizin, 91054 Erlangen, Germany.; ^3^ Department of Physics, Friedrich-Alexander University of Erlangen-Nürnberg, 91058 Erlangen, Germany.; ^4^ Max Planck Institute of Biochemistry, 82152 Planegg, Germany.; ^5^ Department of Biology, Friedrich-Alexander University of Erlangen-Nürnberg, 91058 Erlangen, Germany.

## Abstract

Investigations based on cryo–electron microscopy (cryo-EM), atomic force microscopy, and super-resolution microscopy reveal a symmetric trimer with propeller-like blades for the mechanosensitive ion channel PIEZO. However, a conclusive understanding of its conformations in the cell membrane is lacking. Here, we implement a high-vacuum cryogenic shuttle to transfer shock-frozen cell membranes in and out of a cryostat designed for single-particle cryo–light microscopy (spCryo-LM). By localizing fluorescent labels placed at the extremities of the blades of the mouse PIEZO1 protein in unroofed cell membranes, we ascertain three configurations with radii of 6, 12, and 20 nanometers as projected onto the membrane plane. We elaborate on the correspondence of these data with previous reports in the literature. The combination of spCryo-LM with cryo-EM promises to provide quantitative insights into the structure and function of biomolecular complexes in their native environments without the need for chemical fixation or protein isolation, ushering in a new regime of correlative studies in structural biology.

## Teaser

Cryo–light microscopy resolves the conformational states of the mechanosensitive ion channel PIEZO1 in the native cell membrane.

## INTRODUCTION

**Fig. 1.**
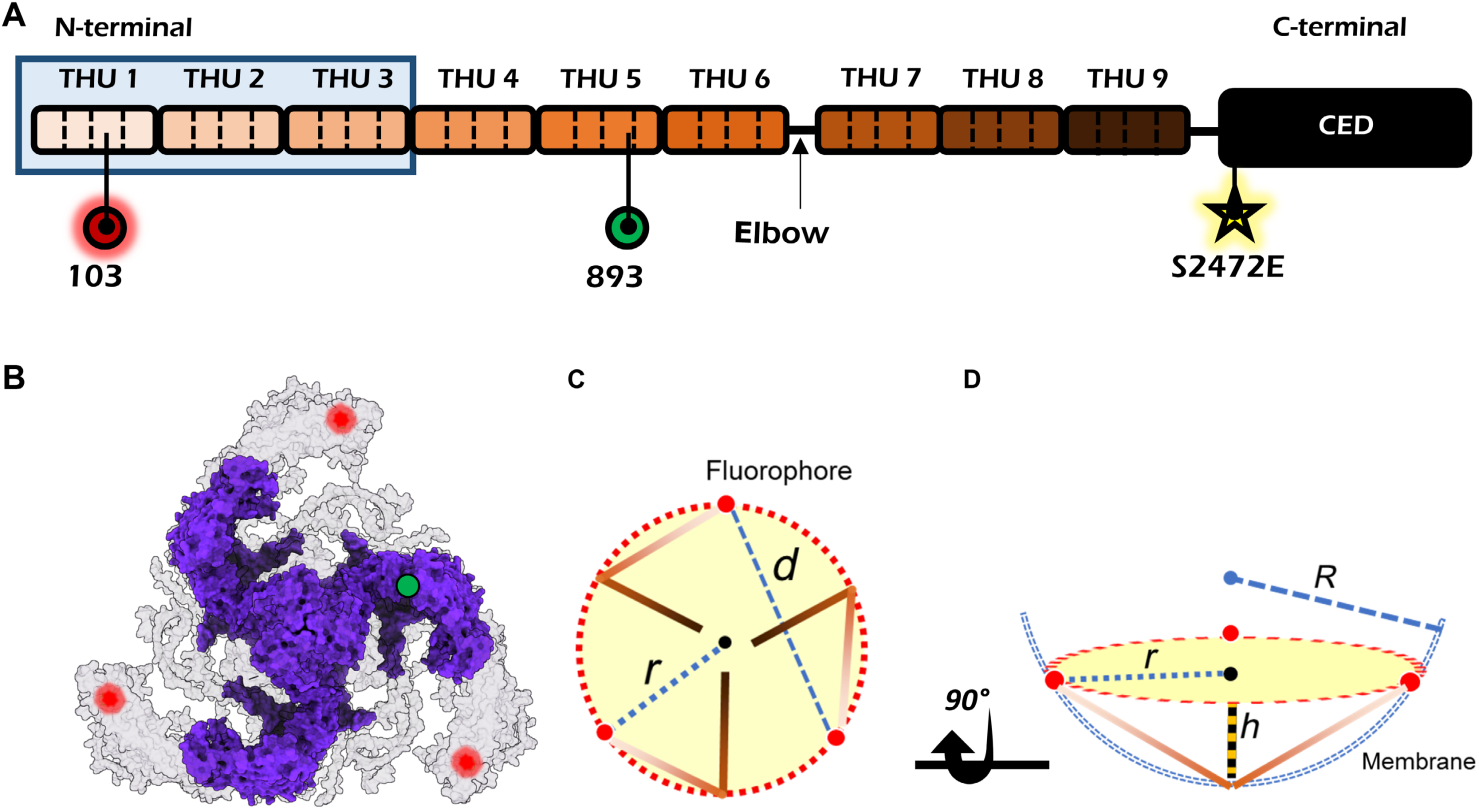
Structure of the mPIEZO1 protein. (**A**) Domain organization of the monomer mPIEZO1 protein composed of nine TM α-helical units (THU), colored in a gradient of brown. Each unit is a bundle of four α helices, separated by black dashed lines. The C-terminal extracellular domain (CED), which forms the core of the channel, is shown in black. The part of the N-terminal shaded in light blue has remained unresolved in EM studies. Positions 103 and 893 mark the labeling positions mentioned in the text. The yellow star marks the approximate prone-activated mutation S2472E. The arrow indicates the elbow region at which the blade structure curves. (**B**) Cryo-EM structure of mPIEZO1 (PDB: 6B3R) in purple docked into the AlphaFold 2 (AF2) predicted structure in gray (E2JF22). Red and green spots show the exact location of labeling position amino acids 103 (our case) and 893 (*17*). (**C**) Schematic top view of the nanodome structure. Brown lines are the blade domains. Blue dashed and dotted lines show the interblade distance (*d*) and in-plane radius (*r*). Our definition of *r* here is with respect to the fluorophore positions and not the outer rim of the PEIZO protein (see fig. S1 for details). (**D**) Side view of (C), illustrating the dome model of the PIEZO protein as explained in ref. (*12*). The blue dashed lines depict the curved membrane with radius *R*.

PIEZO proteins are large transmembrane (TM) mechanosensitive ion channels that are involved in the translation of mechanical forces to biological signals and steer the regulation of various physiological processes (*1*). The PIEZO1 protein is composed of 2547 amino acids (Fig. 1, A and B) and is predicted to form 38 TM α helices (*2*, *3*). The structure was investigated by cryo–electron microscopy (cryo-EM) (*3*–*6*) and atomic force microscopy (AFM) (*7*), and it was found to consist of a symmetric trimer (Fig. 1B), where the C-terminal domain forms the core of the channel (Fig. 1, A and B). The N-terminal domain in each protomer is composed of 36 TM α helices, forming a long blade-like structure whose end points lie on a circle of radius *r* from the core (Fig. 1, A to D; see fig. S1 for a nuanced definition of *r*). Previous studies have revealed that the blade domains curve out the membrane plane into a nanodome structure (*3*, *8*, *9*) (see Fig. 1D), inspiring the hypothesis that the channel is closed in the curved state, and it opens for ion flow when the structure flattens in response to an induced membrane tension (*3*). In other words, curvature plays a central role in mechanosensing (*9*–*13*).

Despite these advances, our understanding of the structure and function of PIEZO proteins remains incomplete owing to the complexity of this protein and various experimental challenges. Much of the existing data have been obtained from isolated proteins reconstituted in detergent or synthetic membranes (*3*, *4*, *6*–*8*). Considering that the details of lipid composition are believed to regulate the PIEZO activity in cells (*1*, *14*, *15*), high-resolution measurements on native cell membranes become indispensable. However, previous efforts in fluorescence microscopy toward deciphering the blade structure were realized in chemically fixated plasma membranes of cells and lacked sufficient spatial resolution and statistics (*16*, *17*). We now exploit an imaging strategy in which we unroof cells, plunge freeze them, and then transfer them with a vacuum-tight cryogenic shuttle to a cryo-super-resolution fluorescence microscope, where single PIEZO proteins are imaged with angstrom precision. The high sensitivity and localization precision of our study sheds light on the structure of the protein, including the extremity of its N terminus, which was not resolved in cryo-EM measurements (Fig. 1, A and B). In the Discussion section of this article, we put our findings in the context of the previous state-of-the-art investigations (*3*–*9*, *16*–*18*). Our approach holds great promise for studying membrane proteins, which are embedded in the challenging surroundings of the cell membrane—an environment that is difficult to access using other structural biology techniques.

## RESULTS

### Cryo–super-resolution microscopy on vitrified specimens

Angstrom resolution in structural studies requires the elimination of all thermal and functional motion that would smear spatial information. Room-temperature super-resolution microscopy achieves this via chemical fixation (*19*, *20*), which generally includes harmful substances to the cells and might result in artifacts and distortions of the biomolecular structure (*21*–*23*). To circumvent this complication, electron microscopists have developed vitrification of biological samples via shock-freezing (*24*), where water molecules are nearly instantaneously frozen into an amorphous ice state (vitreous ice), thus preserving the hydrated environment (*23*, *25*, *26*). This method has also recently been used in correlative cryo-EM and cryo–light microscopy (Cryo-LM) studies, where the latter served as annotation whereas the former delivered high-resolution images (*27*–*31*). However, the technique has not been used for obtaining super-resolution optical images of individual biomolecules (*31*). Here, we use plunge-frozen samples on commercial transmission electron microscopy (TEM) grids to directly obtain cryogenic fluorescence images of individual biomolecules with angstrom precision (*32*).

**Fig. 2.**
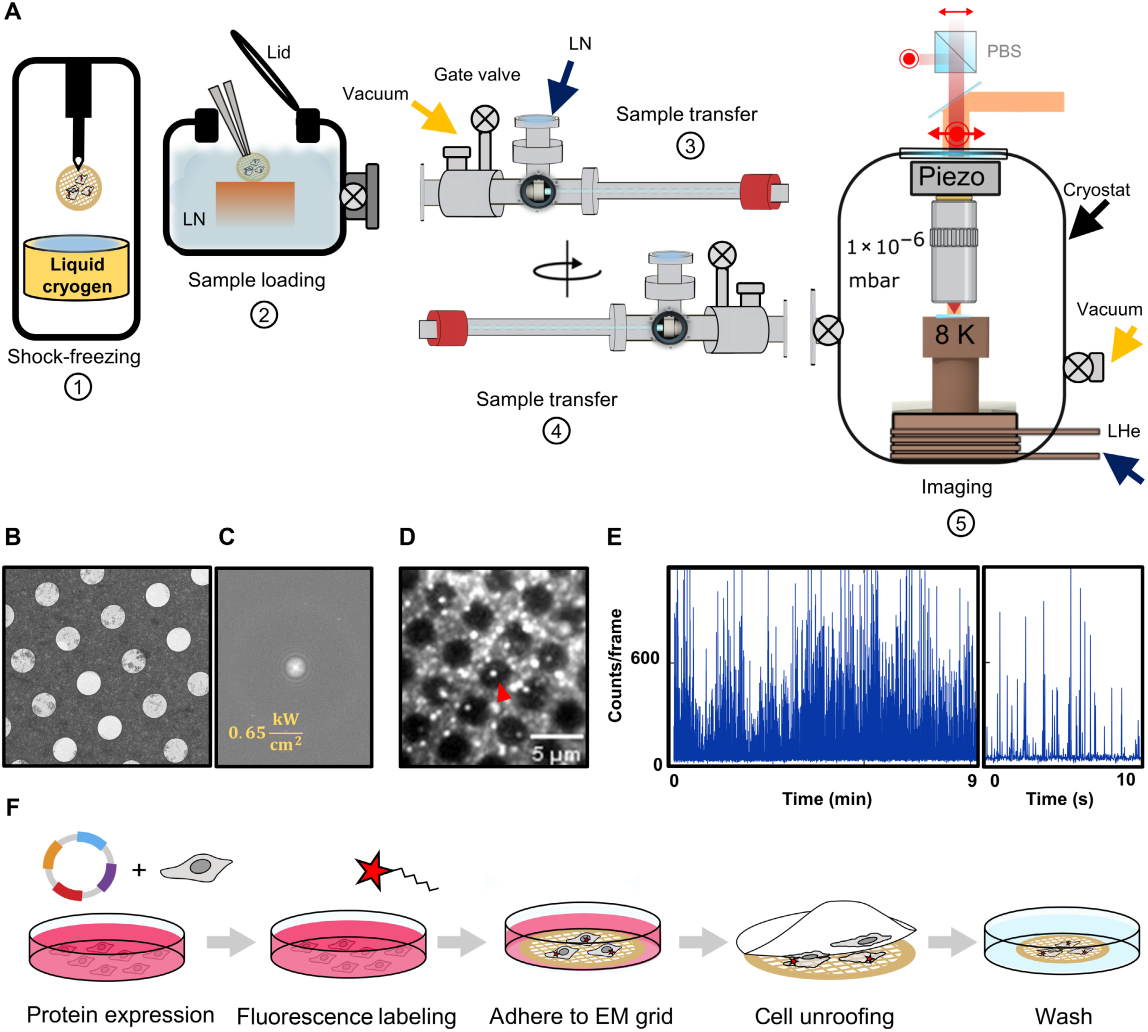
Imaging the mPIEZO1 protein in the near-native cell membrane. (**A**) Schematic view of the five major steps in the workflow. For details, see text, movie S1, and ref. (*32*). We used a polarization-resolved detection scheme to resolve several fluorophores with angstrom precision, exploiting their fixed dipole orientation at low temperature (*37*, *43*). (**B**) Overview images at lower magnification. The hole size in the TEM grid is 2 μm. (**C**) Power spectrum associated with the high-magnification (0.85 Å/pixel) micrographs within the UF holes of TEM grids using cryo-EM following their exposure to laser intensities (0.65 kW/cm^2^). (**D**) Fluorescence image of a vitrified aqueous solution of ATTO647N recorded on a carbon mesh TEM grid. The arrow indicates a single dye molecule sitting within the hole for photophysical characterization. (**E**) An exemplary intensity time trace of a dye molecule indicated in (D) shows large off-times and short on-times suitable for super-resolution imaging. The right panel shows a close-up from the left panel. (**F**) Overview of the pipeline used for cell unroofing and sample preparation.

To achieve near-native imaging of TM proteins, we constructed a shuttle system inspired by the design in ref. (*33*), allowing us to transfer vitrified samples in and out of the cryogenic optical microscope with negligible condensation and devitrification. Figure 2A sketches our principal workflow (see also movie S1), which is described in detail in ref. (*32*). First, TEM grids carrying the target biological sample are plunge-frozen in a liquid cryogen to achieve vitreous ice conditions using a dedicated commercial unit (step 1). Next, the shock-frozen sample is mounted onto a custom-designed copper cartridge within a liquid nitrogen (LN) chamber with low humidity (step 2). Then, the chamber is closed and pumped on to reach low vacuum (~0.1 to 1 mbar). In a following step, the copper sample cartridge is transferred to a shuttle chamber under high vacuum (10^−6^ mbar) and at LN temperature after it is flanged onto the sample loading chamber (step 3). Then, a flange is released under closed gate valve conditions and the shuttle is subsequently flanged onto the cryostat that operates under high vacuum (1 × 10^−6^ mbar) (step 4). By opening the gate valve again, the copper sample cartridge can be transferred to the cold stage of the microscope at liquid helium temperature (step 5). We have tested the viability and stability of the transfer process and the optical microscope by inserting temperature sensors along the path and have shown that the sample remains well below the devitrification temperature during the transfer steps (*32*). The measured temperature of the sample itself equilibrated at ~8 K.

To verify that the environment allows efficient removal of the heat generated by laser illumination, we vitrified pure aqueous samples on several TEM grids (UltrAufoil R2/2 200 mesh) and exposed each to varying laser powers (0 to 1 kW/cm^2^) in the cryostat for a few hours. The samples were subsequently retracted from the cryostat and investigated by cryo-EM to examine the presence of devitrified ice (*32*). Figure 2 (B and C) depicts a micrograph of a TEM grid illuminated at 0.65 kW/cm^2^ (B) together with its associated power spectrum (C). Here, the occurrence and intensity of crystalline ice diffraction rings are used to assess the presence of unwanted crystalline ice (*34*, *35*). We found no notable ice devitrification [see Methods and details in ref. (*32*)].

Fluorescence microscopy at liquid helium temperatures also brings about the decisive advantage that photochemistry and photobleaching are reduced, leading to orders of magnitude more collected photons per fluorophore (*31*). By exploiting the stochastic photoblinking of individual fluorescent labels and sequentially localizing each individual dye with few angstrom precision, it has been shown that different components of a single protein can be resolved (*32*, *36*, *37*). Moreover, the three-dimensional (3D) orientation of each structure can be deciphered in a manner analogous to single-particle cryo–electron microscopy (spCryo-EM). Previous demonstrations used protein structures embedded in a polymer (*36*, *37*). Figure 2 (D and E) shows that the blinking photophysics remains favorable in a vitrified matrix [see movie S2 and details in ref. (*32*)].

### Optical imaging of PIEZO1 at angstrom precision

We imaged three fluorescent dyes placed on mPIEZO1 proteins. To probe the blade conformational state, we used a previously generated DNA construct (*16*) that incorporates an unnatural amino acid *trans*-cyclooct-2-en-l-lysine (TCO*K) at position 103, close to the edge of the unresolved part of the blade (Fig. 1, A and B). The protein construct was expressed in COS-7 cells because these cells adhere strongly and spread on the substrate. Following protein expression, the cells were labeled specifically at position 103 using the small organic fluorophore Pyrimidyl-Tetrazine-AF647 (see figs. S2 and S3). As plunge-freezing is most effective for a sample thickness less than 1 μm (*24*, *26*), we followed a protocol (*38*, *39*) to unroof the cells, leaving only their basal membranes intact on the TEM grid (see Fig. 2F). This technique generally keeps the cell cytoskeleton connected to the cell membrane (*17*, *39*–*41*). It has been shown to maintain Ca^2+^-dependent exocytosis activity (*39*) and to be suitable for protein structure determination using cryo–electron tomography (cryo-ET) (*42*). By labeling the actin filaments of the unroofed cell, we confirmed their presence across the cell membrane (see Methods and fig. S4). We therefore assume that the lateral membrane tension is preserved upon unroofing.

**Fig. 3.**
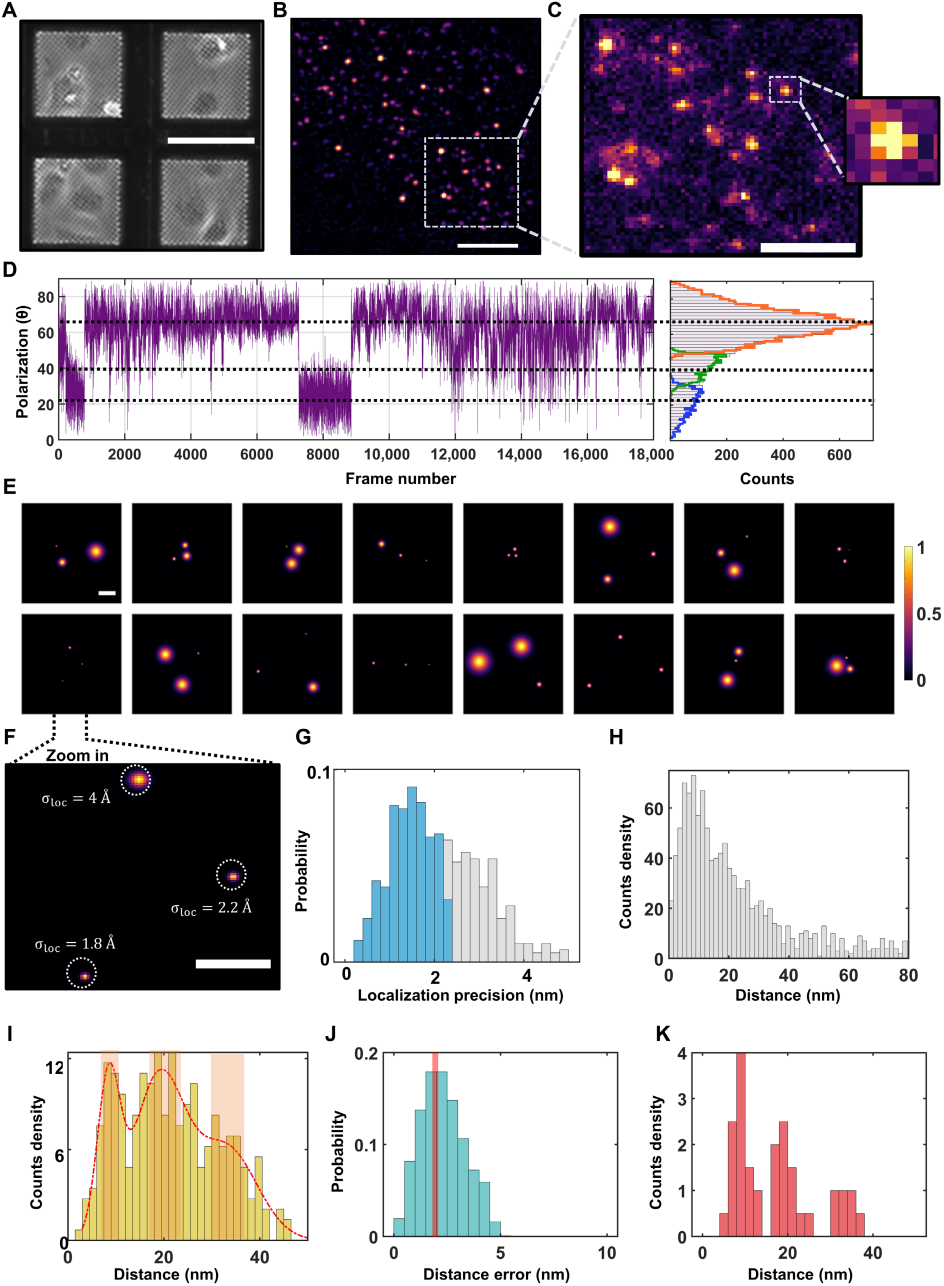
spCryo-LM imaging and analysis of the mPIEZO1 protein. (**A**) Bright-field image of COS-7 cells adhered to UltraAUFoil-2C TEM grids. Scale bar, 100 μm. (**B**) Fluorescence image taken by our cryogenic optical microscope from one of the mesh squares shown in (A). The image is median filtered with a kernel size of 5 pixel by 5 pixel. Scale bar, 10 μm. (**C**) Distribution of fluorescence spots (raw image) across a grid region of 15 μm by 15 μm from (B). Inset shows a close-up of one PSF with a pixel size of 227 nm. Scale bar, 5 μm. (**D**) Exemplary polarization time trace extracted from one of the detected PSFs. The histogram in the right panel shows the polarization time trace decomposed to three states (see Methods). (**E**) 2D resolved images generated from particles with three polarization states. The spots denote the location of the fluorophores, and their widths represent the uncertainty of each localization event. Color bar indicates the normalized probability of fluorophore position. Scale bar, 10 nm. (**F**) A close-up of a particle indicated in (E) demonstrates angstrom localization precision. Scale bar, 5 nm. (**G**) Histogram of localization precisions from 615 particles. The blue area indicates particles with localization precision better than ~2 nm. (**H**) Pairwise distance histogram. (**I**) Histogram of maximum side lengths from each particle. The red curve shows a fit based on a Gaussian mixture model (see also fig. S11). The highlighted regions (light red) indicate selected data for visualizing the aligned 2D images. (**J**) Estimated error of the measured distance, using error propagation (see Methods). The median value indicates an error of 2.3 nm. The red line marks the criterion for filtering the maximum distance histogram in (I). (**K**) Maximum distance histogram in (I) after filtering to include only distances with an error below ~2.2 nm.

Figure 3A shows exemplary bright-field microscopy images of several regions of a TEM grid carrying cells, and Fig. 3B displays a fluorescence image from part of a grid field. In Fig. 3C, we present a close-up image of a 15 μm–by–15 μm region from (B), revealing a sparse distribution of fluorescent spots. The low density of PIEZO proteins in the membrane facilitates their distinction from each other within the diffraction limit. It thus follows that each spot, corresponding to a point-spread function (PSF) of the optical system represents up to three fluorophores. Photophysics characterization of PIEZO proteins labeled with Pyrimidyl-Tetrazine-AF647 showed fast blinking events, with an average off-to-on ratio of ~10, and an average photon count rate of ~5000/s, suitable for high-resolution imaging (fig. S5 and movies S3 and S4). As a validation check, we also imaged labeled nontransfected cells to assess the autofluorescence signal and unspecific binding of fluorescent molecules (fig. S6 and movie S5).

Given that, in the frozen state, the emission dipole moment of each dye molecule is randomly fixed in space, the three fluorophores on a protein can also be distinguished through polarization-selective detection (*37*, *43*). In the current study, we chose PSFs that displayed three polarization states only (see Fig. 3D), amounting to a total of *N* = 615 analyzed particles. This was obtained from ~50 fields of view (FOVs; 48 μm by 70 μm; see Fig. 3, A and B), which approximately accounts for 50 to 150 cells in total. The yield of such particles is influenced by several factors such as successful unnatural amino acid incorporation and labeling efficiency (see Methods). Figure 3E shows a mosaic of 2D projections from 16 proteins (see fig. S7 for more particles). The size of each spot in these images signifies the precision with which it was localized. Figure 3F presents a close-up of the fluorophore positions from one protein, where all three were localized with angstrom precision. In Fig. 3G, we plot the distribution of the localization precision for the total population, with a median at 1.3 nm and an average precision of 1.4 nm per protein (see fig. S5 for the precision on the *x* and *y* axes separately). Selection of cases with average localization precision better than ~2 nm for further analysis reduced the number of particles to 378. We note that the localization precision and the statistics could be further enhanced by extending the sample imaging time because photobleaching is nearly suppressed (fig. S5).

The overall distribution of the distances *d* between neighboring fluorophores projected onto the imaging plane gives rise to the gray histogram in Fig. 3H. The 3D-to-2D projection smears the major side lengths of a triangle toward shorter distances. To ascertain whether the observed broad distance distribution arises from diverse triangles with unequal side lengths or from several subpopulations of equilateral triangles with different side lengths, we aligned all the projections about their centers of mass (fig. S8) and performed in-plane rotations (fig. S8). The outcome yielded a symmetric Y-shaped pattern (see figs. S8 and S9), providing strong evidence for near-symmetric equilateral conformations. However, the classification of different subpopulations within the broad histogram of Fig. 3H is not straightforward.

**Fig. 4.**
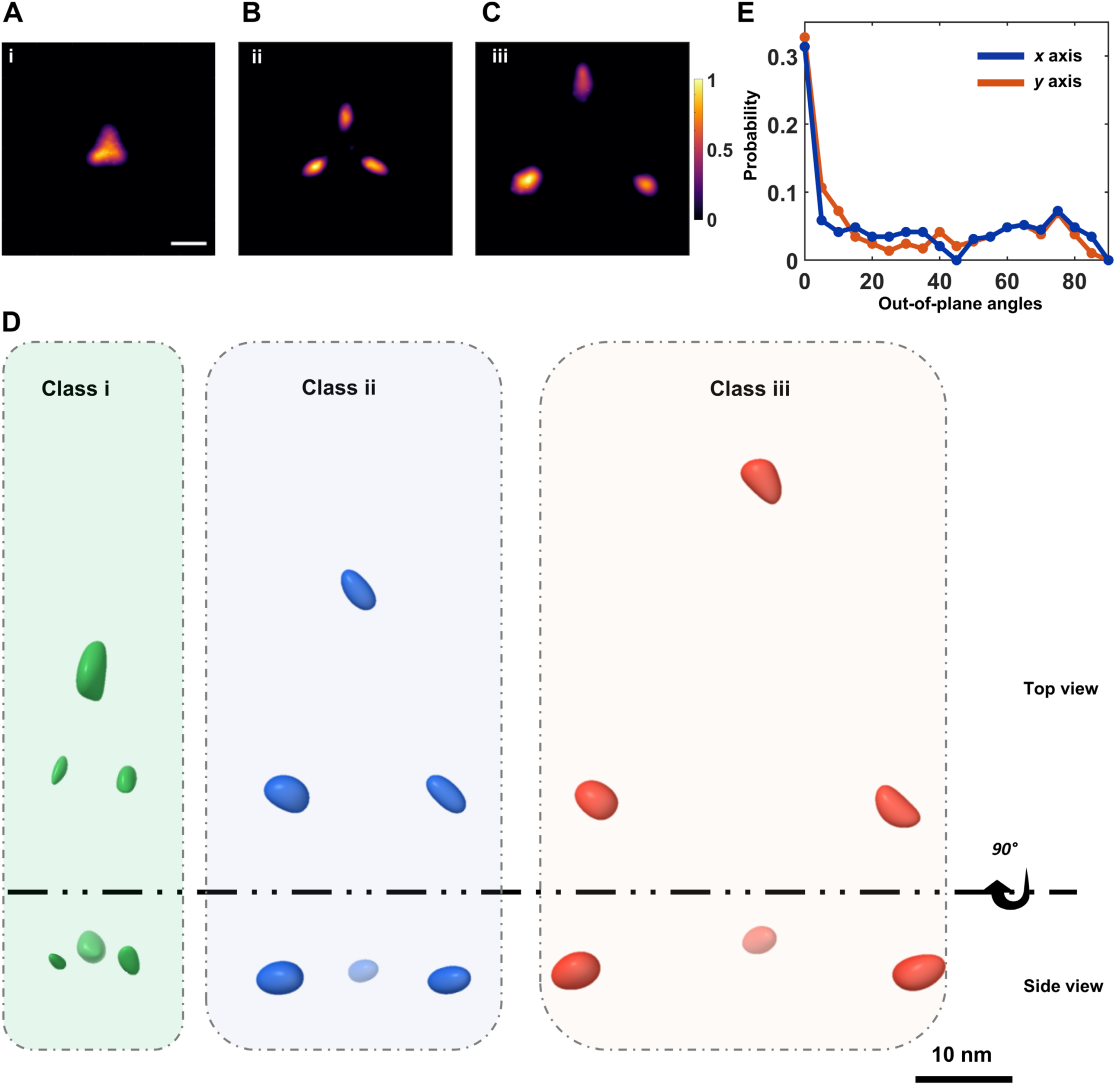
Dissecting the structural conformations of the mPIEZO1 protein. (**A** to **C**) 2D projections after aligning all measured particles through rotations. Panels [(A) to (C)] show the subpopulations for the three distances identified in Fig. 2: *d* = 9 (i), 19 (ii), and 34 (iii) nm, establishing three classes of conformations (see also figs. S7 to S11). The final images were filtered against a lower probability (below 0.5) of outlier projections. The smear of the localization spots is influenced by the localization precision and protein orientations [see (E)]. Scale bar, 10 nm. (**D**) Top-view and side-view projections of the 3D reconstructed volume of each class. Spheroids in green, blue, and red indicate the localization uncertainties (see movies S6 to S8). We note that no *C*_3_ symmetry was imposed, i.e., the resulting feature emerged from the raw data. (**E**) Probability of the out-of-plane orientation of the particles in the range of [0° to 90°], as estimated based on a simulated annealing algorithm (see Methods). The blue and orange curves indicate the probability of the out-of-plane angles around the *x* axis (pitch) and the *y* axis (roll), respectively. As can be seen from the plot, the particles appear with the highest probability close to 0°, i.e., lying in the observation and membrane plane, whereas the probability of out-of-plane angles is lower (see fig. S7).

To address this difficulty, we exploited the fact that for randomly oriented equilateral triangles, one of the projected side lengths always remains very close to the triangle side length: In the case of no localization error, the projection falls within a small range bounded by d and $\;\frac{\sqrt{3}}{2}d$ (see fig. S10). Figure 3I displays the resulting histogram, revealing peaks at *d* ~ 9, 19, and 34 nm, termed classes i to iii, respectively, determined from a fit by a Gaussian mixture model (fig. S11). Thus, by taking the maximum side length from each measured protein, we minimize the smearing effect of 2D projections. We confirmed that the existence of three major peaks is not sensitive to binning parameters (see fig. S10). In addition, in a recent study, we showed that this approach can also capture the major side length in nonsymmetric triangles (*32*). Moreover, simulations indicate that the amount by which each subpopulation is broadened is close to the localization precision limit (fig. S10). To estimate the error in the extracted values of *d*, we propagated the localization errors for each dye (see Methods). As shown in Fig. 3J, the median of the resulting distribution corresponds to 2.3 nm. Figure 3K shows that, if we filter the data to keep cases with distance errors < 2.2 nm and select polarization trajectories with a signal-to-noise ratio larger than ~3.5, the three peaks become resolved more clearly. Figure 4 (A to C) visualizes the 2D projections corresponding to the three distance groups (see also fig. S8). The same results can also be obtained if we simply select the particles that belong to the central regions of each identified peak in Fig. 3I (marked by shaded bands).

Having established the three most probable equilateral triangle side lengths, next we used a classification pipeline to include the data from all measured proteins to assess the fractional weight of each class. Here, we first generated 2D projection templates for equilateral triangles as model system. We classified each measured projection based on a template matching score between 0 and 1 to one of the side lengths of 9, 19, and 34 nm (see figs. S7 and S11), yielding subpopulations of 21, 51, and 28%, respectively. Next, we used the classified projection to arrive at their 3D volume using a reconstruction algorithm (*37*, *44*) with a resolution of 4 to 8 Å as estimated from the Fourier shell correlation (FSC) analysis (*45*) (see fig. S11 and movies S6 and S8). In Fig. 4D, we present the top (i) and side (ii) views of the projections from the resulting 3D reconstructions. Figure 4E shows that, as expected for a TM protein, proteins mostly lie in the membrane plane. Here, one should also bear in mind that a native plasma membrane is not fully flat but can contain regions of local curvature, where proteins are oriented out of the imaging plane.

### Structural conformation of the open-prone mPIEZO1-S2472E mutant

**Fig. 5.**
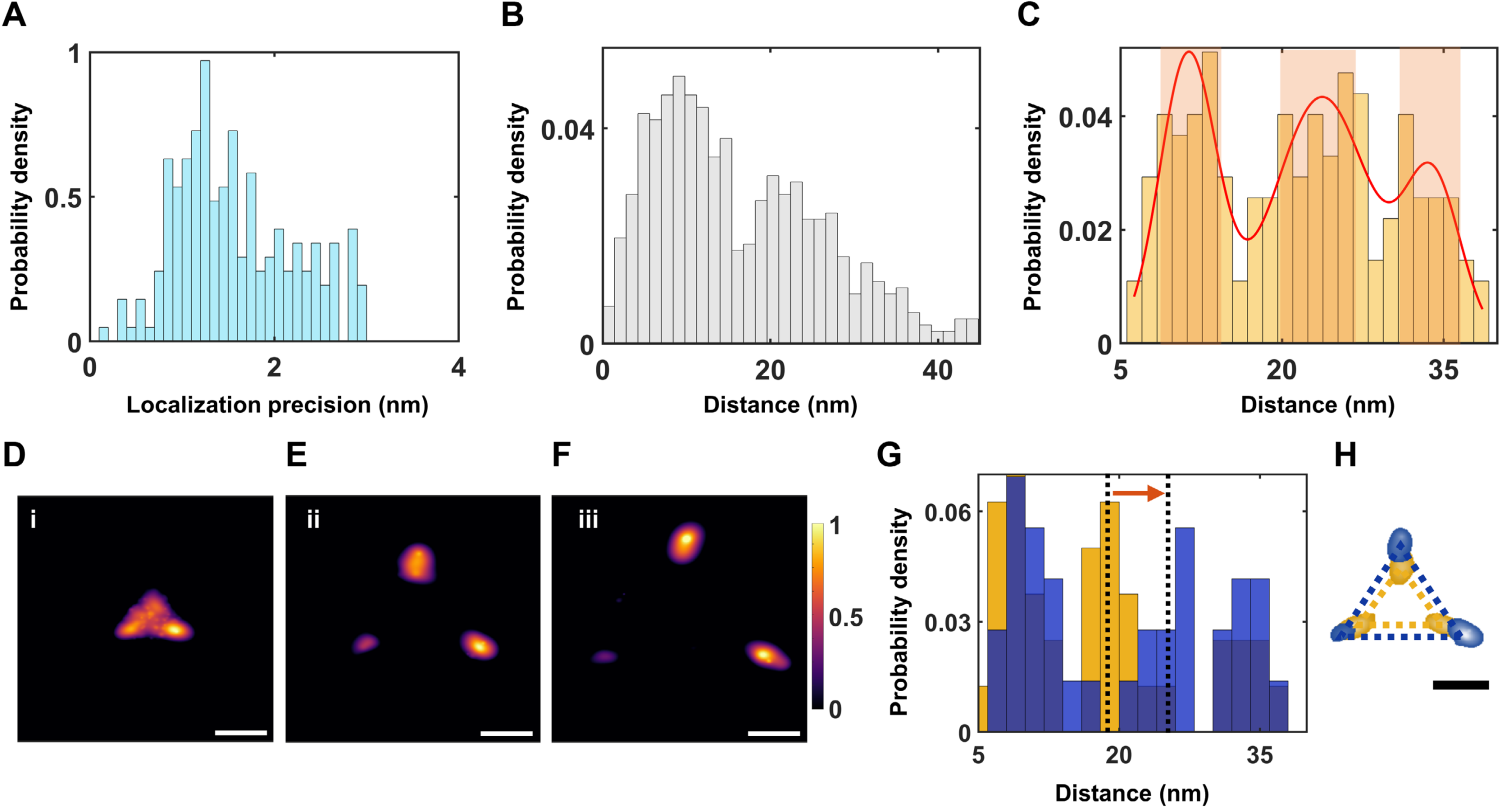
spCryo-LM imaging and analysis of the prone-activated mPIEZO1-S2472E mutant. (**A**) Histogram of localization precisions. (**B**) Histogram of pairwise distances between fluorophores (206 particles). (**C**) Histogram of maximum side lengths from each particle (bin size is 1.47 nm). The red curve shows a fit based on a Gaussian mixture model, yielding three components based on the Akaike information criterion. The highlighted regions (light red) indicate the selected data for visualizing the aligned 2D images in (D) to (F). (**D** to **F**) 2D projections after aligning all measured particles through rotations. The panels show the subpopulations for the three distances identified in (C): *d* = 11 (i), 24 (ii), and 34 (iii) nm. The final images were filtered against a lower probability (below 0.5) of outlier projections. The smear of the localization spots is influenced by the localization precision and protein orientations. Scale bars, 10 nm. (**G**) Overlay of the filtered maximum distance histogram of WT mPIEZO1 (mustard; from Fig. 3K) and the mutant mPIEZO1-S2472E (blue). The data show a clear shift of class ii conformation from 19- to 24-nm interblade distance. The bin size is 2 nm. (**H**) Overlay of the 2D aligned projection of the WT mPIEZO1 (Fig. 4B; mustard) and mPIEZO1-S2472E mutant (Fig. 5E; blue).

In a recent study (*18*), an intermediate open-channel state of PIEZO1, termed prone-activated state, was generated via mutagenesis. The authors revealed through electrophysiological experiments that, by using a single-point mutation of the amino acid serine to glutamic acid (S2472E) at TM helix 38 of the C-terminal extracellular domain (see Fig. 1A), PIEZO1 can be stabilized in a conducting state, where the channel is open-prone for ion flow. We also followed this strategy using the same plasmid construct to probe the structural-functional relationship of the blade domain (Fig. 1). In this study, we selected particles with three fluorophores from the spCryo-LM data with localization precision better than 3 nm and maximum interblade distance below 45 nm, accounting for 206 particles in total obtained from ~40 FOVs. In Fig. 5A, we plot the distribution of the localization precision for the total population, with a median at 1.3 nm (see fig. S12 for exemplary projection images). Figure 5B plots the overall pairwise distance histogram, which is clearly different from that obtained in Fig. 3H. To inspect this further, we also plot the maximum side length from each measured protein, as explained before. Figure 5C displays the resulting histogram, revealing three major peaks at *d* ~ 11, 24, and 34 nm, termed classes i to iii, respectively, as determined from a fit by a Gaussian mixture model. Figure 5 (D to F) visualizes the ensemble-averaged 2D projections corresponding to the indicated three distance groups in Fig. 5C, after rotating and aligning the 2D projections. Again, this result indicates the effective classification of the data and reveals near-symmetric equilateral triangles with expected side lengths. We observe shifts in the histograms of class i (from 9 to 11 nm) and class ii (from 19 to 24 nm) toward a larger distance, respectively (Fig. 5, G and H). Although the change in class i population is within the estimated median distance error of ~2.3 nm, we consider the conformational change to class ii to be substantial (Fig. 5, G and H). This is in line with the observations of cryo-EM studies, indicating a partially flattened blade domain and a shift of ~2.5 nm with respect to the curved mPIEZO1 structure (*18*).

## DISCUSSION

A notable result of the first cryo-EM studies of mPIEZO1 in detergent (*3*–*6*) was that the blades project out of the plane. To investigate the mechanical interplay between the PIEZO protein and its surrounding membrane, several groups have reconstructed mPIEZO1 in synthetic liposomes (*3*, *7*–*9*). These works have verified that the blade domain can curve the membrane into a dome-shaped structure when the C-terminal extracellular domain faces the inside of the liposome, acquiring an out-of-plane curvature *R* ~ 10 nm (see Fig. 1D). In addition, measurements on the configuration of the C-terminal extracellular domain faces the outside of the liposome realized the flat conformation of the mPIEZO1 protein in synthetic liposomes with *r* = 14 (*8*). As shown in Fig. 1 (C and D), top-view imaging measures distance *d* between certain blade locations and can yield *r*, whereas imaging membrane cross-sectional cuts can provide insight into the out-of-plane curvature of the protein and that of the membrane. The quantities *r* and *R* can be connected via the relation $R=\frac{r^{2}+h^{2}}{2h}$ , based on a spherical surface that reaches height *h* (*12*). We note that, to capture the variation of the blade ends, we consider *r* to mark the fluorophore positions and not the outer rim of the PIEZO protein (see fig. S1 for details).

AFM-based experiments could demonstrate the transition of mPIEZO1 from the curved to flat state in real time when reconstituted in a supported lipid bilayer (SLB) (*7*), although the direct link between the flat state and channel activity is still missing. Although it is commonly assumed that the channel operates through a two-state mechanism (curved and flat), some studies have observed three states. For example, AFM-based experiments found two main populations with *r* ~ 11 and 17 nm and other minor population at *r* ~ 22 nm (*7*). Moreover, a very recent cryo-EM study (*18*) generated a prone-activated state of mPIEZO1 via mutagenesis in a detergent environment and identified three dominant conformations with *r* ~ 10, 12, and 12 nm and estimations of *R* ~ 14, 32, and 110 nm, termed curved, intermediate, and fully flat, respectively.

Another study developed a model based on membrane elasticity theory to determine the shape of the mPEIZO1 dome in an asymptotically flat membrane and predicted *R* ~ 42 ± 12 nm (*9*, *12*), which is significantly larger than the estimated value of *R* ~ 10 nm in liposomes (*7*, *8*). To examine the validity of the abovementioned findings for proteins in the native cell membrane, further questions have to be addressed: Are there other important contributions from cellular compartments such as the cell cytoskeleton, and how is the nanodome structure affected by the complexity of the plasma membrane? Although most studies have established that force from lipids is sufficient to tune the conformational changes of PIEZO (*9*, *11*, *13*), it is important to investigate these phenomena in the native environment. Several experimental efforts have thus examined mPIEZO1 in cells but using chemically fixed samples. In a first work, the PIEZO protein was prepared in unroofed red blood cells and labeled at position 893 (see Fig. 1) using antibodies. By using STED (stimulated emission depletion) imaging, a distance of ~25 nm between the fluorophores was reported (*17*) whereas the antibody-based labeling likely overestimated the actual distance. In addition, these measurements had low resolution and low statistics. Recently, by incorporating an unnatural amino acid at position 103 (see Fig. 1) and using a small organic label, the interblade domain of mPIEZO1 in human embryonic kidney (HEK) 293 cells was resolved with higher resolution using minimal photon fluxes (MINFLUX) microscopy (*16*). Under rest conditions and no treatment, the authors reported a single broad population centered at ~25 nm for the interblade distances. This changed to ~20 nm after treating the cell with a specific drug (GsMTx-4), which affects the protein-membrane interface, and to ~35 nm in a hypotonic solution. However, because of limited statistics (less than ~40 particles overall) and low resolution (~10 nm), configuration subpopulations were not resolved.

As the position of amino acid 103 on the blade is not resolved by cryo-EM studies, we compare our data with the fully predicted structure model of mPIEZO1, generated by the artificial intelligence program AlphaFold 2 (AF2) (*46*) and its most recent version AF3 (*47*) (fig. S13). Here, it should be kept in mind that, as the trained datasets mainly stem from isolated protein models, exact structural prediction of protein structures in the native membrane environment is not fully reliable. For example, AF2 (*46*) and AF3 (*47*) predicted different structural models of mPIEZO1 (fig. S13). We summarize the outcome of the previous studies (*3*, *7*–*9*, *16*–*18*) in table S1.

To place our measured blade distances in the context of the previous reports, we converted the measured blade distances into the in-plane radius *r* (Fig. 1C) following the relation $r=\frac{d+1.5\pm 2.3}{\sqrt{3}}\;$ nm, where *d* signifies the distance between the blades at position 103. The value 1.5 in the numerator is the approximated distance from position 103 to the edge of the blade estimated on the basis of the AF2 model (E2JF22), and 2.3 nm signifies our experimental distance error (Fig. 3J). Following this procedure, we obtain *r* ~ 6 (class i), 12 (class ii), and 20 (class iii) ± 1.3 nm for the three observed configuration classes discussed above.

**Fig. 6.**
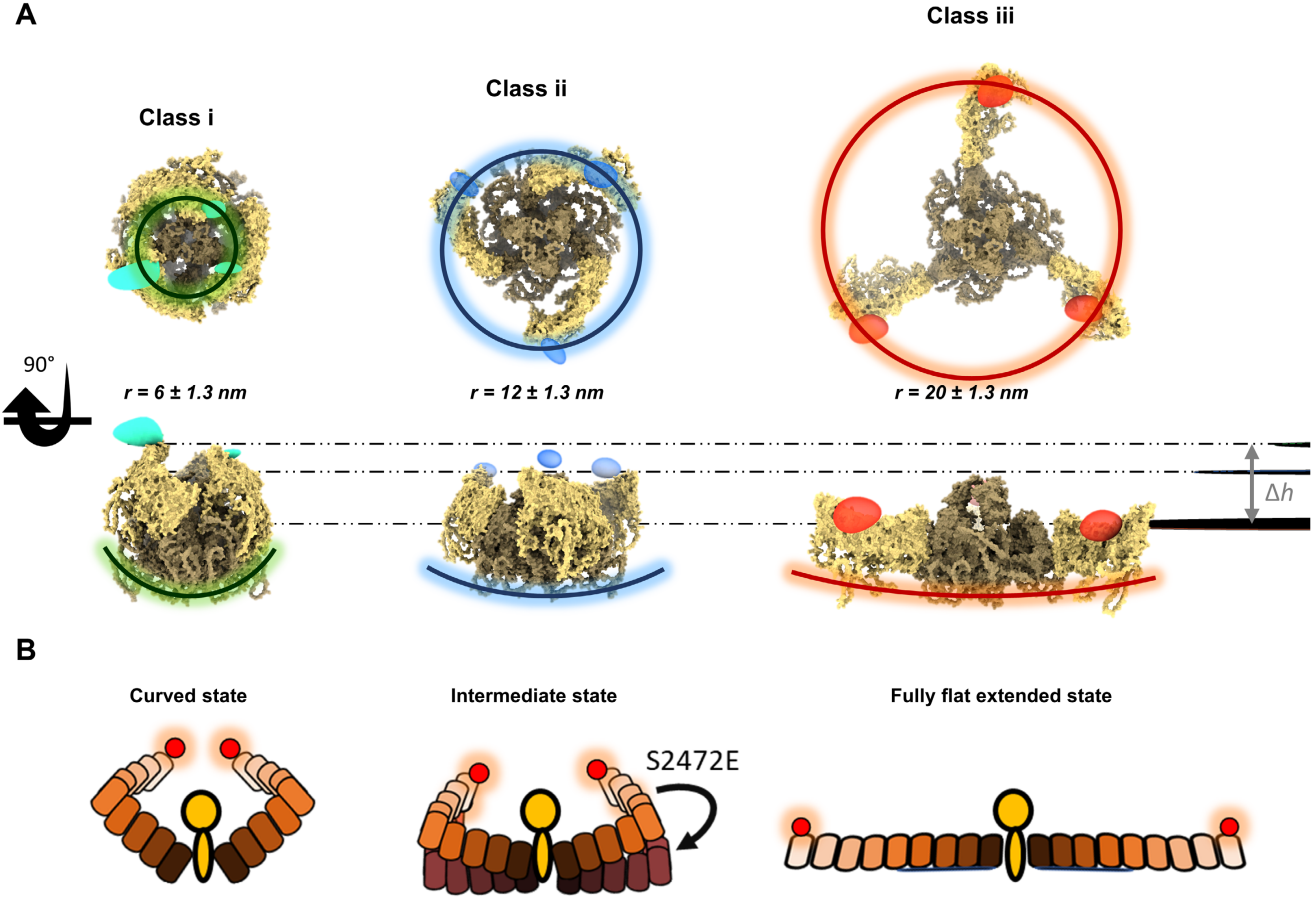
Structural modeling of the mPIEZO1 conformations. (**A**) Structural models based on AF2 and AF3 obtained after rigid-body rotation (class i and class iii) and AF2 structure (class ii), which match our experimentally measured distances (see Methods, fig. S13, and movie S9). Spheroids highlighted in green, blue, and red indicate the 3D resolved volume using the classified 2D projections, for classes i, ii, and iii, respectively. Their position matches the structural model (see movie S9). Horizontal dashed lines indicate the planes of fluorophore locations. (**B**) Schematics of the model deduced from our data, showing the highly curved, intermediate, prone-activated mutant and fully flat extended state of the blade domain. The difference in height of the ends of the blades between class iii and class i [see dashed lines in (A)] amounts to ~5.5 nm.

Our findings for the class ii (*d* = 19 ± 2.3 nm and *r* = 12 ± 1.3 nm) are in excellent agreement with the predictions *d* = 19.5 nm and *r* ~ 12 nm of a structural model based on AF2 (E2JF22) (Fig. 6A, fig. S13, and movie S9). We note that this agreement applies to the interblade distance only and not the height of the blade, which dictates the radius of curvature. In the case of the S2472E mutant, we find *d* ~ 24 nm and *r* ~ 15 nm for class ii, also in close agreement with the cryo-EM results of *d* ~ 23 nm and *r* ~ 14 nm obtained for the flatter blade conformation [Protein Data Bank (PDB): 8IXO] of proteins reconstituted in detergent (*18*) if we account for the unresolved part of the blade (see fig. S13 and Methods).

For class i, our measured *d* and *r* values are considerably smaller than previous findings (*3*, *7*, *8*). AF3 suggests that the blades are rotated further clockwise toward the center of the protein to yield *d* = 13.5 nm and *r* ~ 8 nm (fig. S13). Comparison of these data with our measurements *d* = 9 ± 2.3 nm and *r* = 6 ± 1.3 nm suggests that, in addition to the clockwise movement of the blades toward the center of the protein, they must also move out of the membrane plane. To examine this hypothesis, we considered the region between TM24 and TM25 (Fig. 1A and fig. S13) as an “elbow” for the PIEZO1 blades (*3*, *7*) and conducted rigid-body rotations of the blade arm out of the plane around the identified elbow region (see Methods). This simple structural assumption can describe a small upward movement of the blades, ~25°, yielding a highly curved state (see Fig. 6A, fig. S13, and movie S9).

In the case of the largest distance between the blades in class iii, our data (*d* = 34 ± 2.3 nm and *r* = 20 ± 1.3 nm) indicate a larger structure than the flat conformation (PDB: 7WLU) of mPIEZO1 reconstituted in a lipid model system (*d* ~ 26 nm and *r* ~ 15 nm) obtained in cryo-EM (*8*) (see fig. S13). Here, we hypothesize that the blades move by an additional in-plane counterclockwise rotation around the previously mentioned elbow region, thus generating a straighter shape and occupying a larger area than what has been reported previously. By rotating the blade structure around the elbow, we found that an in-plane counterclockwise rotation of ~50° very closely represents the measured distance of *d* ~ 34 nm and *r* ~ 20 nm, resulting in a fully flat and extended PIEZO state (see Fig. 6A, fig. S13, and movie S9). Our data for this conformation match well the reported averaged value of ~34 ± 8 nm by MINFLUX for cells treated with hypotonic extracellular solution, which causes the blade domain to stretch (*16*).

We cannot directly assess the side-view radius of curvature *R* of the PIEZO-membrane system as discussed in previous works (*9*, *18*). However, in Fig. 6B, we examine the variations in height between the three scenarios based on the structural models that follow from our measurements (see also Fig. 1D). Our data on mPIEZO1 wild type (WT) and the S2472E mutant show that the interblade distance remains unchanged for classes i and iii, consistent with the view that these are the two extreme scenarios of mPIEZO1 curvature and expansion. Thus, if we consider *h* = 0 for the (nearly fully) flat case of class iii, the height difference to the highly curved state of class i becomes Δ*h* ~ 5.5 nm, in agreement with the range of *h* ~ 0 to 5 nm proposed in the literature (*7*, *18*) (see fig. S13 and movie S9). From our structural model and the maximum estimated height of class i, we then calculate *R* = 6 ± 2 nm, indicating mPIEZO1 in a highly curved membrane topography (Fig. 6). We note that these findings do not contradict the theoretical estimation of ref. (*9*), which were based on asymptotically flat model membranes.

As there is some uncertainty regarding the *h* value of class ii, we calculated *R* over the estimated range of *h* = 0 to 5 nm (fig. S13), resulting in a median value of *R* = 30 nm with 95% confidence interval from 26 to 35 nm (Fig. 6B). Thus, we conclude that class ii conformation (51% of the population) must be an intermediate state with a flatter structure. If we consider class ii–S2472E mutant, for the same *h* interval of 0 to 5 nm, we obtain a median *R* = 40 nm with a confidence interval from 35 to 47 nm. This result indicates that the blades must be flatter in the mutant than in the WT. Furthermore, we attribute class iii conformation to the nearly fully flat state of mPIEZO1 with a completely unbent blade domain. We note that, although R=∞ for *h* = 0, a slight deviation to *h* = 1 nm would imply *R* ~ 200 nm. In summary, our data are consistent with the most recent observations of three conformational states of mPIEZO1 reported by cryo-EM (*18*). Our data suggest that mPIEZO1 adopts a highly curved conformation, an intermediate with partially flat but bent blades state, and lastly a configuration with fully flat extended blades (see Fig. 6). In particular, by capturing the edges of the blade domain, our data show that the blade extremities conform to a well-defined arrangement whereas previous studies fell short of this observation, which they partly attributed to the flexibility of the blade domain (*16*, *18*).

Angstrom precision in single-particle cryo–light microscopy (spCryo-LM) allowed us to reveal three major conformational states of the mPIEZO1 protein in its native cellular membrane. To achieve these results, we introduced an experimental approach to circumvent the use of in vitro purification methods such as detergent, liposome, or nanodisc (*48*–*50*), as well as harmful chemical fixation protocols, which have commonly been used in high-resolution studies (*19*, *20*). Our results provide a direct and quantitative evidence for two extreme scenarios of the blade expansion and curvature, which were not clearly resolved by other methods. We believe a key advantage in our approach is the high signal-to-noise ratio of the localization procedure from each individual measured protein, thus reducing the need for averaging processes that are currently necessary in cryo-EM analyses. With its high localization precision and robust statistics, our methodology has enabled sorting and classification of individual proteins into different configuration states in the complex environment of the cell membrane, which had previously remained elusive to other optical super-resolution microscopy efforts (*16*).

Our data indicate that mPIEZO1 can occupy a more highly curved state in the cell membrane than what has been shown in liposomes and from AF3 predication. Moreover, we revealed the structural insight on blade unbending in the flat state, where they rotate in an anticlockwise manner to occupy a larger area. We anticipate that the observed unbending might contribute to the gating mechanism, e.g., by stabilizing the channel opening or acting under extreme stretching conditions of the cell membrane. Overall, our data suggest that the blades undergo a spiral rotational motion from a fully extended state in a flat conformation toward a state with a small in-plane radius and blades bent to a highly curved configuration out of the membrane plane. This finding is in line with the predications of AF2 and AF3 and assumption made in an earlier cryo-EM study (*6*) (Fig. 6B).

Further improvements in the labeling strategy, e.g., by simultaneous measurement of the blades with respect to the C-terminal domain of the mPIEZO1 protein, will allow one to resolve the curvature of the protein in the membrane. In addition, repeated fast laser-induced vitreous ice melting and reverification can be used to probe conformational changes on the same protein (*51*). Furthermore, in situ scanning tips can be used to interrogate the response of the protein-membrane system to mechanical stimulation in the cryogenic microscope (*52*–*54*). Findings from spCryo-LM will also be helpful for improving the AF models, which have thus far been mainly trained data from isolated proteins. A particularly exciting vision is to realize correlative measurement with cryo-ET (*55*, *56*), where spCryo-LM provides PIEZO conformational states, whereas cryo-ET delivers information on the ultrastructural context of the membrane curvature.

## METHODS

### Cryogenic sample transfer

After plunge-freezing, we first mount the sample onto a dedicated sample cartridge. Next, the sample is transferred using a high-vacuum transfer shuttle operated at LN to a high-vacuum precooled liquid helium cryostat that houses the optical microscope. The process can also be executed in the reverse order to retrieve the grid for subsequent analysis and correlative imaging. Further details of the high-vacuum cryogenic transfer shuttle and characterization of the system can be found in movie S1 and in ref. (*32*).

### Preparation of vitrified fluorescent molecules

Carbon TEM grids (R3.5/1, 200 mesh or others) were plasma cleaned with 14% plasma power for 10 s inside a Faraday cage (Diener, Pico 500 W). UltraAuFoil TEM grids with 2-nm carbon were plasma cleaned at 24% plasma power and 20-s incubation time. ATTO647N-maleimide fluorophores (150 pM) were prepared in 25 mM Hepes buffer solution. A 3.5-μl fluorophore solution was applied onto the grid and blotted after 30 s of incubation time, using Vitrobot (IV, Thermo Fisher Scientific) with standard Vitrobot blotting paper (47000-100, PLANO GmbH) and the following parameters: 1-s blotting time, −10 force, and 0 waiting time at 100% humidity and 4°C. After plunge-freezing, the samples were transferred into our optical microscope as described in detail in Fig. 2 and in ref. (*32*). We then allowed the sample to relax thermally and mechanically from 80 K down to 8 K for ~1 to 2 hours before we start data acquisition.

### Vitreous ice determination by cryo-EM

Vitrified samples were loaded into a 300-kV Titan Krios G2 (Thermo Fisher Scientific), equipped with a K3 direct electron detector + Bioquantum energy filter (Gatan). Grid atlases of all samples were montaged at low magnification (34x) using SerialEM (*57*). Subsequently, grid positions around the center and very far away from the center were imaged at eucentric height at an intermediate magnification (2250x, corresponding to ~3.9 nm/pixel) to gain an overview of the local ice thickness and hole coverage. At the center of ice-covered holes, a high-magnification exposure at 105kx (corresponding to 0.85 Å/pixel) with a total dose of around 60 e/Å^2^ was recorded. The power spectra of such high-magnification exposures were then qualitatively examined for the presence and intensity of crystalline ice rings. Here, the occurrence and intensity of crystalline ice diffraction rings, with their characteristic band between 6 and 30 Å, can generally be used to assess the presence of unwanted crystalline ice (*34*, *35*). For example, after inspecting the power spectra (Fig. 2, B and C), we find that pre-exposure (0.65 kW/cm^2^) has no noticeable devitrification (Fig. 2B). For further details on the effect of laser intensity on the quality of vitreous ice, please refer to the detailed method procedure in (*32*).

### Mouse PIEZO1 sample preparation

The coding sequence of mPIEZO1 protein, including an amber stop codon at position 103, was obtained as a gift from the A. Patapoutian group at The Scripps Research Institute and described in detail in ref. (*16*). We generated the S2472E mutant using standard site-directed mutagenesis on the same mPIEZO1 coding sequence described above. The plasmid pNEU-hMbPylRS-4xU6M15, which encodes the tRNA that incorporates the unnatural amino acid pNEU-hMbPylRS-4xU6M15, was a gift from I. Coin (Addgene plasmid no. 105830) (*58*). COS-7 cells (DSMZ, catalog no. ACC 60) were grown in Dulbecco’s modified Eagle’s medium (Gibco, Invitrogen) supplemented with 10% fetal calf serum (Gibco, 10270-106, lot 2307596) in a humidified atmosphere at 37°C and 5% CO_2_. For transfection, the cells were grown to 60 to 70% confluency in a T25 flask (Thermo Fisher Scientific). The media of the cell were then exchange to fresh media containing 500 μM TCO*K unnatural amino acid (Sirius Fine Chemicals SiChem GmbH, catalog no. SC-8008). The cells were then transfected using Lipofectamine 3000 Transfection Reagent (Invitrogen, L3000001) as described in the manufacturer’s protocol, with a 2:1 ratio of the tRNA:mPIEZO1 plasmid at a final DNA content of 6 μg per T25 dish, and incubated for ~36 hours. Two to 4 hours before harvesting the cells, we exchanged the media with fresh media, which do not contain any transfection reagents or unnatural amino acid to allow the cell to release the excess unnatural amino acid. The cells were then washed several times with Dulbecco’s Phosphate Buffered Saline (DPBS) buffer, detached, pelleted, and resuspend in fresh media. A total of ~1.5 × 10^6^ cells were then moved into a 1.5-ml Eppendorf tube and incubated for 10 min with fresh media contacting 0.01% gelatin and fibronectin (2.5 μg/ml) solution as a blocking reagent. The cells were then pelleted and incubated with fresh media containing 4 μM Pyrimidyl-Tetrazine-AF647 (Jena BioScience, CLK-102) and put on a shaker for 20 to 30 min at room temperature and blocked from ambient light. The cells were moved to a 15-ml Falcon tube and were then pelleted twice and exchange with fresh media (~10 ml) to allow the washing of excess dye. Last, the cell pellet was resuspended with 2-ml fresh media and transferred to a 3.5-cm μ-Dish (Ibidi) containing eight UltrAuFoil QF R2/2 TEM grids with 2-nm carbon on top (UF, S373-7-UAUF-2C, Plano GmbH) facing upward. The cells were allowed to adhere onto the grid for overnight. The UF TEM grid with 2-nm carbon on top was prepared by plasma cleaning at 24% plasma power for 15- to 20-s incubation time inside a Faraday cage (Diener, Pico 500 W), followed by dipping in 70% ethanol and DPBS. The grids were then coated with 0.02% gelatin containing fibronectin (5 μg/ml) for 2 hours at 37°C to enhance cell adhering before the unroofing process. The grids were placed on top of a 20-cm-diameter coverslip, which is smaller than the inside diameter of the 3.5-cm μ-Dish, to allow sliding the TEM grid in an angle for easier handling and picking without bending.

### Cell unroofing

The following day, the cells were washed with warm DPBS containing Ca^2+^ and Mg^2+^ (Corning, 21-030-CV). The cells were then dipped into 2:1 Milli-Q with cytosol-like buffer (CLB) containing 20 mM Hepes, 130 mM K gluconate, 5 mM NaCl, and 2 mM MgCl_2_ (pH 7.4) for 30 s. Six microliters of undiluted CLB was added onto the grid followed by putting the grid on top of filter paper (Whatman filter paper 2, catalog no. 1002-055), where the cells side facing the paper (see Fig. 2F). The buffer was allowed to be absorbed by the paper, and the grid was picked once the absorbent disk buffer stopped to grow. The cells were then dipped in clean CLB, which contains 1:200 protease inhibitor (PI) cocktail (Sigma-Aldrich, catalog number: 539134). We then put the cell dish on a shaker for 30 min to wash the cytosolic content out. The cells were then washed x3 with fresh CLB without PI. Some of the grids were inspected to confirm the presence of unroofed cell via membrane staining or F-actin staining (see fig. S4). Next, we loaded the cell into the Vitrobot and applied 3.5 μl of fresh CLB buffer on the cell side. The grid was blotted using the following parameters: 0 force, 3.5-s blotting time, and 0 waiting time at 100% humidity and 22°C. To avoid further unroofing or detachment of the membrane, we used a Teflon sheet facing the side of the membrane and standard blotting paper on the other side. This protocol was adopted on the basis of a recent work (*38*).

### Optical setup

All experiments were performed in a cryogenic microscope that is built around a Janis ST-500 flow cryostat and operates at liquid helium temperature (*37*, *43*). The microscope is mechanically stable, with a measured drift of 5 nm/min on average (*32*). In addition, the temperature on the cold finger is stable at 8.23 K irrespective of the laser power. The optical path is the same as in our previous work and can be found in refs. (*32*, *37*, *43*). Briefly, samples are loaded onto a cold finger and imaged by a 0.90–numerical aperture objective (MPLAN 100x, Mitutoyo), which is mounted in vacuum, onto two separate electron-multiplying charge-coupled device cameras (Andor iXon) in a polarization-resolved configuration. For fast acquisition rates, the FOV was set to 211 pixel by 313 pixel with a pixel size of 227 nm. The laser intensity used in all experiments was set to ~0.65 kW/cm^2^, and images were recorded with 14-ms exposure times, more than five times faster than the typical off-time. Therefore, we are able to capture individual bursts and minimize the probability of overlapping emission from two or more fluorophores in a single frame. For each FOV, we collected a total of 50,000 to 100,000 frames depending on the experiment.

### Image analysis

We analyzed raw image stacks from two polarization channels with custom-written MATLAB software as described in detail in two of our recent works (*37*, *43*). However, instead of the median filtering approach, we used the band-pass filter algorithm (*59*) to detect the PSFs, with a low pass value of 0.3 and a high pass value of 3. The band-pass filtered images were then further filtered using a threshold value of 10. Polarization time traces of multifluorophore particles are then analyzed in more detail by a routine that is based on an unsupervised signal fitting approach, the DISC algorithm (*60*), using Bayesian information criteria with an alpha value of 1, and then refined using a 2D Gaussian mixture model classification approach that exploit the polarization and coordinates simultaneously to find the number of polarization states per PSF. The fit of the traces was then inspected visually. We note that this trace is not a continuous time trace, but it rather represents the on-states of the fluorophores during the recording, i.e., off-times were omitted. The number of identified polarization states (dipole orientations) in each PSF corresponds to the number of fluorophores per PSF/protein particle as the dipole orientation at 8 K of each fluorophore is random but fixed (*37*). This, in turn, allows us to annotate each fluorophore over time and localize it with high precision beyond the diffraction limit by clustering their coordinates accordingly (*37*). A 2D super-resolved image is then reconstructed by assigning a 2D Gaussian function to each localized polarization/fluorophore with a width given by the respective localization precision. The longer the occupancy of the polarization state, the higher is the localization precision (*37*). These super-resolved images demonstrate different projections of the protein molecules within the sample. We note that two localizations may appear as a single spot for a given particle orientation in the image plane if they overlap within the measurement precision. 2D images are then subject to further analysis such as distance measurements, classification, and 3D reconstruction, as described below.

### Cell and particle statistics

Typically, when cells reach ~80 to 90% confluency on the TEM grid before unroofing, we observe one to three cells occupying each mesh grid (fig. S4). Some cell detachment might occur during the unroofing process. After unroofing, we are no longer able to optically identify cell regions (see fig. S4). This prevents a precise quantification of the remaining cells at the final preparation stage. However, on the basis of our imaging parameters (50+ FOVs, each covering 48 μm by 70 μm, approximately one-third of a 100 μm–by–100 μm grid mesh) and the initial cell density (one to three cells per FOV), we conservatively estimate that our analysis includes between 50 and 150 cells in total. The yield of PIEZO proteins detected with all three fluorophores remains limited due to several factors: (i) labeling efficiency, (ii) photobleaching, (iii) the yield of unnatural amino acid incorporation, and (iv) PIEZO assembly on the membrane. Consequently, we exclude PIEZO complexes with one to two fluorophores from our analysis; completely unlabeled proteins are, by default, not detectable.

### Single-molecule 2D image analysis of mPIEZO1

After generating the 2D resolved images from all the particles (*N* = 615), we picked the ones with average localization precision below 2 nm (*N* = 378) for further analysis (fig. S11). First, we calculated the pairwise distance between the three localizations in each particle, and then we took the maximum side length as explained in Fig. 3. From this result, we identified three major peaks at ~9, 19, and 34 nm, based on a Gaussian mixture model. For the maximum side length histogram, we used a bin size of 1.5 close to the median localization precision of 1.4 nm. We then aligned all the projection on top of each other as they can report on the symmetry and conformations of the mPIEZO1 protein (fig. S8). Here, we use a 2D image template containing three large spots arranged in a triangle shape. The experimental data were then rotated in-plane until the cross-correlation between the experimental image and template is maximized. Then, we summed all rotated projection to identify the symmetry and conformations in the sample. The summed projection indicates a Y-like shape, indicating a distinct near-symmetric trimer conformation with side lengths of 9, 19, and 34 nm (figs. S8 and S9). Next, we classified the image projections using three different 9, 19, and 34 equilateral triangles as a template, following the approach described in ref. (*37*). Of the 378 particles, we were able to identify 61 particles that match class i (8 nm), 145 class ii (19 nm), and 78 class ii (34 nm), and 94 remained unclassified as they show a large distance beyond the PIEZO protein dimension (larger than 43 nm) (see fig. S11). Plotting the sum of the aligned projections for each identified class separately confirms proper classification of the particles as we could identify their conformation in the 2D plane. The mPIEOZ1-S2472E mutant data were processed exactly as described above.

### Distance error estimation

The errors on the measured distances were calculated in an error propagation manner as follows: $\sqrt{\sigma_{1}^{2}+\sigma_{2}^{2}}$ , where $\sigma_{1,2}$ indicate the localization precision of positions 1 and 2, respectively. The latter quantities are calculated as follows: $\sigma_{1,2}=\sqrt{\frac{\sigma_{x}}{\sqrt{N}}*\frac{\sigma_{y}}{\sqrt{N}}},$ where $\sigma_{x,y}$ indicate the SD of the position in the *x*-*y* plane, respectively, and *N* is the number of localizations per position.

### Estimation of particle orientation

We used a simulated annealing algorithm to find the orientation of our particles in the 2D space. Here, we used a triangle model with side lengths of 9, 19, and 34 nm that is allowed to explore all possible rotation angles [yaw (*z* axis), pitch (*x* axis), and roll (*y* axis)] and then find the 2D projection that maximizes the similarity between the projected and the experimental images using 2D cross-correlation (2DCC). To prioritize smaller pitch and roll angles (out-of-plane orientation) while allowing free rotation in yaw (in-plane orientation), we introduce an angular penalty term in the objective function as follows

Objective=−2DCC+ϑ(pitch+roll)

where ϑ is the penalty weight. After estimating the particle orientation, we calculate the probability distribution of angles along the *x*, *y*, and *z* axes.

### Tomography and 3D reconstruction

Here, we selected the particles that were fitted properly to the simulated 2D projections. The 2D maps of the particles were then normalized, and the median of the full widths at half maximum was taken as the localization uncertainty for all particles, to obtain a spherical volume. The 2D projection was then fed into the 3D reconstruction subspaceEM algorithm (*44*). Here, we used an elliptical Gaussian as an unbiased initial structural model. We run the algorithm using the default settings for 100 runs. The 2D maps were constructed using a grid size of 200 by 200 with a pixel size of 1.5 Å for class i and 250 by 250 with a pixel size of 2 Å for classes ii and iii. The 3D volumes were then processed, fitted to the crystal structures/maps of the dye location, and edited using ChimeraX (*61*). The FSC curves were computed using the freely available FSC server, provided by the PDB in Europe website (https://ebi.ac.uk/pdbe/emdb/validation/fsc/). Here, we divide the 2D image datasets into two equally datasets and calculate their 3D reconstruction similar to as described above but using the 3D reconstructed volume as an initial model. Then, we align the two reconstituted volumes and calculate the FSC to detriment the resolution based on half-bit criteria (*45*). We note that no *C*_3_ symmetry was imposed in our 3D reconstruction. The resulting structures emerged naturally from the raw data.

### Blade modeling

We used a pivot point in the elbow region (THU 6, TM22, and amino acid number 1014) that is close to a large predicted loop region. All the amino acids from the N-terminal side until the identified pivot point were rotated from 0° to 180° as a rigid body in the plane or out of the plane with a rotation step size of 5°. The generated structures were then inspected via interblade distance measurement to find the one that matches our experimental data (see fig. S13 and movie S9). Class i, the curved state, was built on the basis of the AF3 structural model. Here, beside the maximum movement of the blades toward the center, which yield *d* = 13.5 nm, we moved the blades further out of the plane by ~25° to match our experimental data. Class iii, the fully flat state, was initially built using a chimera of the partially resolved cryo-EM model (PDB: 7WLU) and the AF2 model. The blades were then rotated in the plane, from which we found the one that matches our experimental data. For class ii, we used AF2 prediction (E2JF22) without any modifications as it matched our in-plane radius. Given the assumption that the blade domain does not unfold, we assume that class i and class iii structural models are unique solutions. However, for class ii, there are several scenarios of the blade rotation and height that might yield the measured 19-nm distance. However, on the basis of our radius of curvature estimation, the blades should be flatter than the predicted structure (E2JF22). A similar analysis was performed for the class ii–S2472E mutant. Here, we used the cryo-EM structure (PDB: 8IXO) and extended its unresolved region by incorporating the predicted structure from AF2. This was achieved by aligning the overlapping regions of the blade domain using PyMOL software.

### Data visualization and analysis

All data analysis was done using MATLAB MathWorks software. Some of the figures were plotted using OriginPro 2020. Protein structures and 3D reconstruction volumes were processed using PyMOL v2.4 and ChimeraX v1.7.1.

## Supplementary Materials

### The PDF file includes:

Figs. S1 to S14 Table S1 Legends for movies S1 to S9 References
