# Supplementary material for: Cryo–light microscopy with angstrom precision deciphers structural conformations of PIEZO1 in its native state
Source: Sci Adv. 2025 Aug 20;11(34):eadw4402. doi: 10.1126/sciadv.adw4402 (PMC12366687; doi:10.1126/sciadv.adw4402)
Supplement: Supplementary file 1 — Figs. S1 to S14 Table S1 Legends for movies S1 to S9 References [file sciadv.adw4402_sm.pdf]

Science Advances
AAAS

Supplementary Materials for

Cryo–light microscopy with angstrom precision deciphers structural conformations of PIEZO1 in its native state

Hisham Mazal *et al.*

Corresponding author: Vahid Sandoghdar, vahid.sandoghdar@mpl.mpg.de;
Hisham Mazal, hisham.mazal@mpl.mpg.de

Sci. Adv. **11**, eadw4402 (2025)
DOI: 10.1126/sciadv.adw4402

The PDF file includes:

Figs. S1 to S14
Table S1
Legends for movies S1 to S9
References

Other Supplementary Material for this manuscript includes the following:

Movies S1 to S9

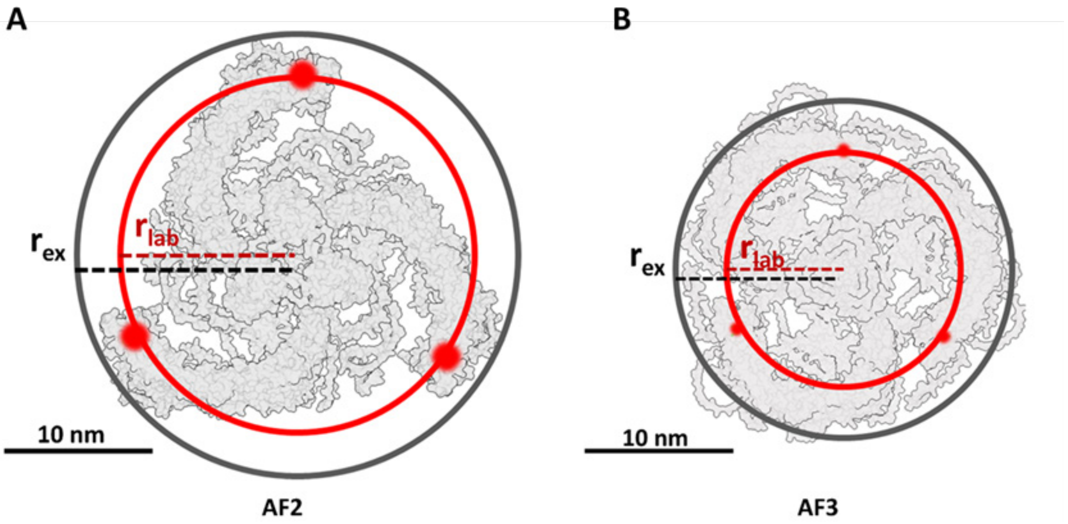

Fig. S1. In-plane radius definition. Comparison of the in-plane radius (*r*) measured directly from the fluorophores' position (r_{label}, red circle) and from the outer rim of mPIEZO1 ($r_{exterior}$, black circle), as shown for two cases based on AlphaFOLD2 (AF2) predication model **(A)** and AlphaFOLD3 (AF3) **(B)**. In our work, we use r_{label}, as it directly imaged, as well as it captures the changes of the blades more accurately. For example, if we consider the in-plane radius based on ($r_{exterior}$) for AF2 and AF3 models, we obtain close values of $r_{exterior}$=~12 nm for AF3 and $r_{exterior}$=~14 nm for AF2, despite their structural differences. In contrast, r_{label} yields ~8 nm for AF3 and ~ 12 nm for AF2. Thus, the structural difference is more pronounced when measured via r_{label}, whereas $r_{exterior}$ underestimates the full curvature of the blade domain.

Labelling control of COS7 cells expressing mPIEZO1 protein.

In order to record single-molecule data with sufficiently low background, one has to find the optimum fluorophore with good labelling efficiency and low nonspecific binding. To do so we have tested three far-red fluorophores with a tetrazine functional group suitable for click chemistry: 1. Tetrazine-ATTO647N (CLK-012-02 Jena Bioscience GmbH), 2. Pyrimidyl-Tetrazine-ATTO-643 (CLK-101, Jena Bioscience GmbH), and 3. Pyrimidyl-Tetrazine-AF647 (CLK-102, Jena Bioscience GmbH). First, we grow the COS7 cells to 70% confluency and then incubate them with the transfection reagents as well as with 500 µM unnatural amino acid trans-Cyclooct-2-en – L - Lysine (TCO*K, SC-8008, SiChem GmbH) as for the normal transfection procedure described in the Methods, however without the plasmid expressing mPIEZO1 protein. After ~36 hrs, the cells were washed and labelled with each of the fluorophores at 4 µM final concentration and incubated for 30 min at 37 °C. The cells were then washed and imaged in bright-field and fluorescence modes to check the labelling background (Fig. S2 A-D). Simultaneously we also labelled cells that had been transfected with mPIEZO1 protein (Fig. S2 E-F)

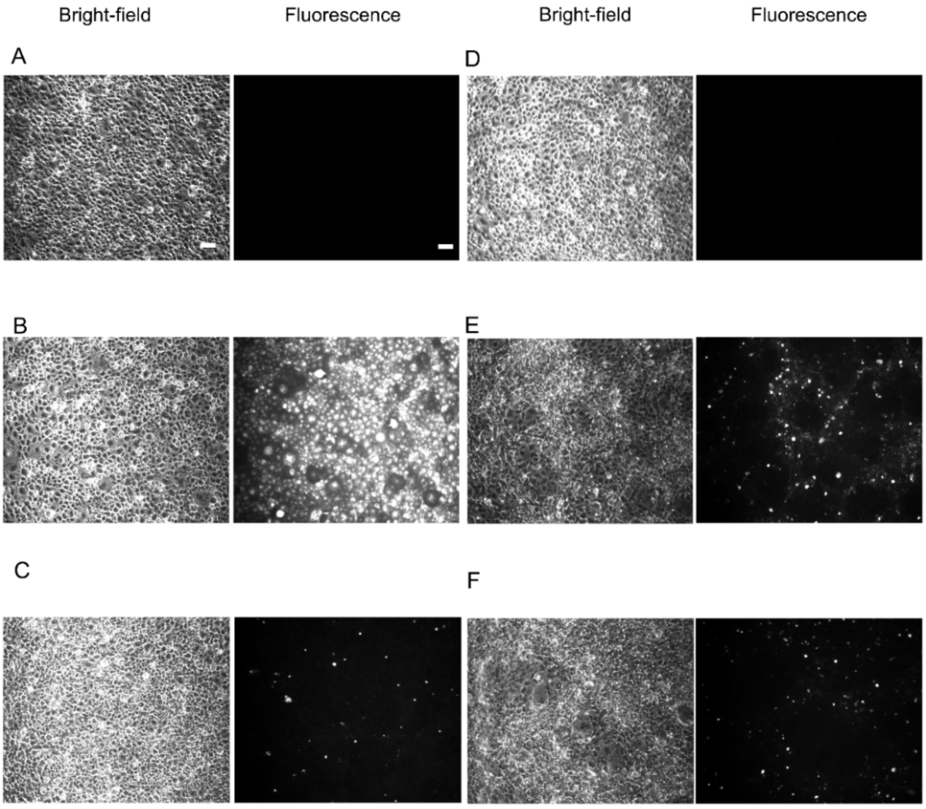

Fig. S2. Labelling control of COS7 cells expressing mPIEZO1 protein. (A) A negative control, which does not include any fluorophore. Left panel: bright-field (BF) image of cells. As expected, the fluorescence image (right panel) shows no signal. **(B)** Cells labelled with Tetrazine-ATTO647N show high background fluorescence signal, indicating high reactivity of non-specific binding. **(C)** Cells labelled with Pyrimidyl-Tetrazine-ATTO-643 show some fluorescence background, indicating medium non-specific binding. **(D)** Cells labelled with Pyrimidyl-Tetrazine-AF647 show no fluorescence signal at all, indicating superior non-specific binding. **(E-F)** Cells transfected with mPIEZO1 protein labelled with Pyrimidyl-Tetrazine-ATTO-643 (**E**) and Pyrimidyl-Tetrazine-AF647 (**F**). In both cases, we see sufficient labelling signal. We opt to work with Pyrimidyl-Tetrazine-AF647 as it shows the minimal background from non-specific labelling. Scale bar in all images is 100 µm. The intensity threshold was set to be the same in all panels except for panel B, which results in signal saturation.

A

B

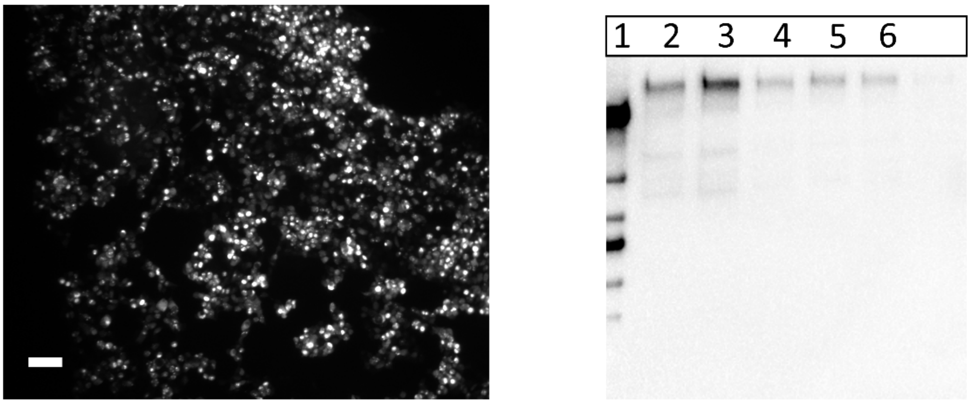

Fig. S3. Expression control of mPIEZO1 protein. To confirm that the unnatural amino acid is incorporated properly and that full-length mPIEZO1 protein is expressed, we transfected HEK293 cells with mPIEZO1 protein as explained in detail for COS7 cells. **(A)** Fluorescence image shows the cells after labelling with Pyrimidyl-Tetrazine-AF647 as described earlier. Scale bar is 100 µm. The cells were then harvested for giant plasma membrane vesicles (GPMVs) production as described in detail in the Ref (*62*). **(B)** We ran Western blot to analyse the mPIEZO protein size using antiHalotag antibody (encoded at the C-terminal domain of the protein). Lane 1 is the Spectra Multiocolor broad range protein ladder (Cat: 26634, Thermofisher), lane 2 is the isolated GPMVs, lane 3 is the same as lane 2 but concentrated using Pierce Protein concentrator PES 50K molecule weight cut-off (Thermofisher), lane 4-5 are further purification steps by utilizing the Twin-Strep-tag on the mPIEZO1 protein using Strp-Tactin XT column (IBA Lifesciences GmbH). The mPIEZO1 protein was observed above the 260 kD band of the ladder, as expected for full length mPIEZO1 (~ 292 kD), indicating full expression.

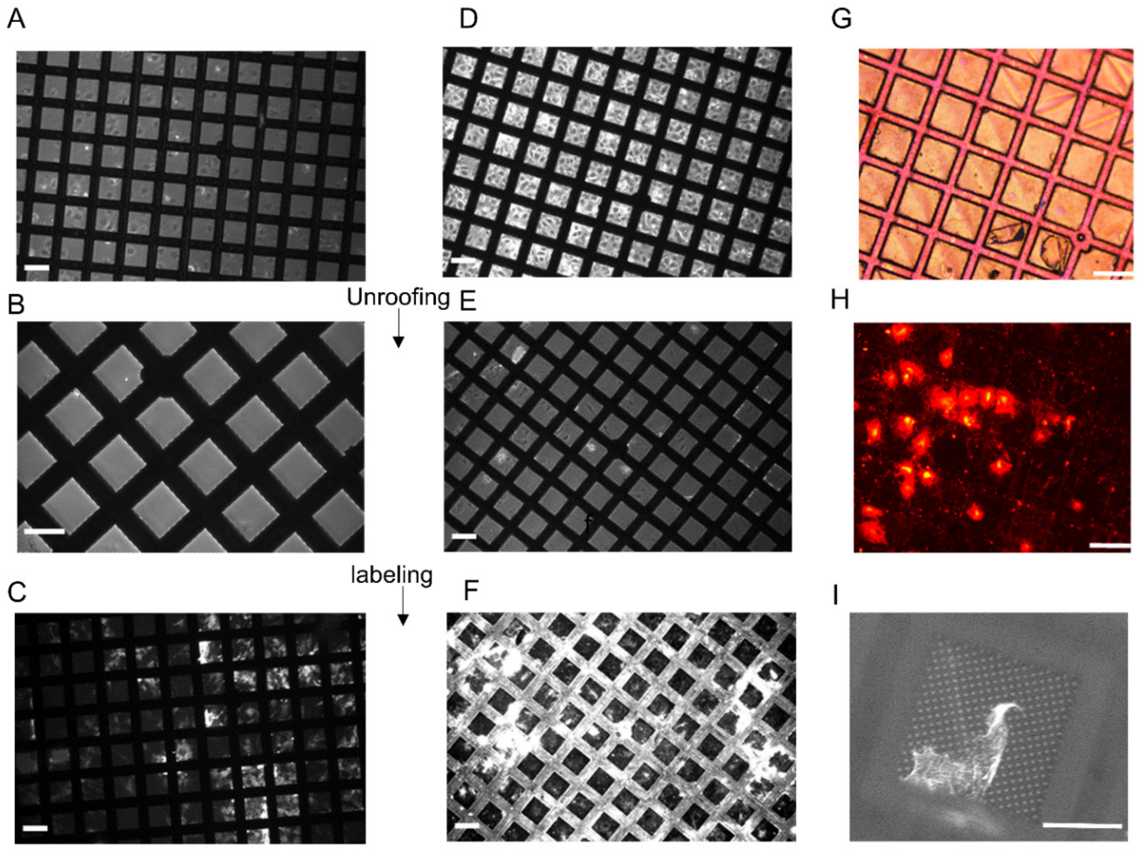

Fig. S4. Unroofing of COS7 cells that express mPIEZO1 protein. (A-C), and (D-F) present two examples of adherent cells on the TEM grid, with different cell coverage densities. After successful labeling of the protein expressed in COS7 cells, we performed a series of washing steps to remove unbound fluorescent molecules. Subsequently, we allowed the cells to adhere on top of an UF TEM grid with 2 nm carbon on top, pre-coated with gelatin-fibronectin, overnight. We validated that the cells indeed adhered and morphologically looked healthy. The adherent cells imaged using transmission bright-field mode (Leica DMIL LED) and look morphologically healthy as one can observe in (A, D). We unroofed the cells using blotting paper, leaving the bottom part of the cell intact on the TEM grid. As on can notice in the transmission bright-field images in (B, E), the grid looked clean, as no cells present there. However, after staining the membrane using carbocyanine dyes (DilC18) for 10 min, one can observe a fluorescence signal across the grid, as presented in (C, F), indicating the presence of thin sheet of membrane. The fluorescence images recorded using the same optical microscope in transmission mode using 520 nm excitation. **(G)** We recoded the same grid as in panel (E), in BF reflection mode using a Leica DM 4000 M microscope, which also shows a clear grid. **(H)** Fluorescence image of the grid mentioned in panel (G) using the same microscope, indicate the presence of cell membrane. (**I)** unroofed cells were labeled with phalloidin-532 for 1 hr, and imaged using the Leica DMIL LED microscope. The image clearly shows the actin filaments across the cell. Scale bar in panels (A-H) is 100 µm. Scale bar in panel (I) is 50 µm.

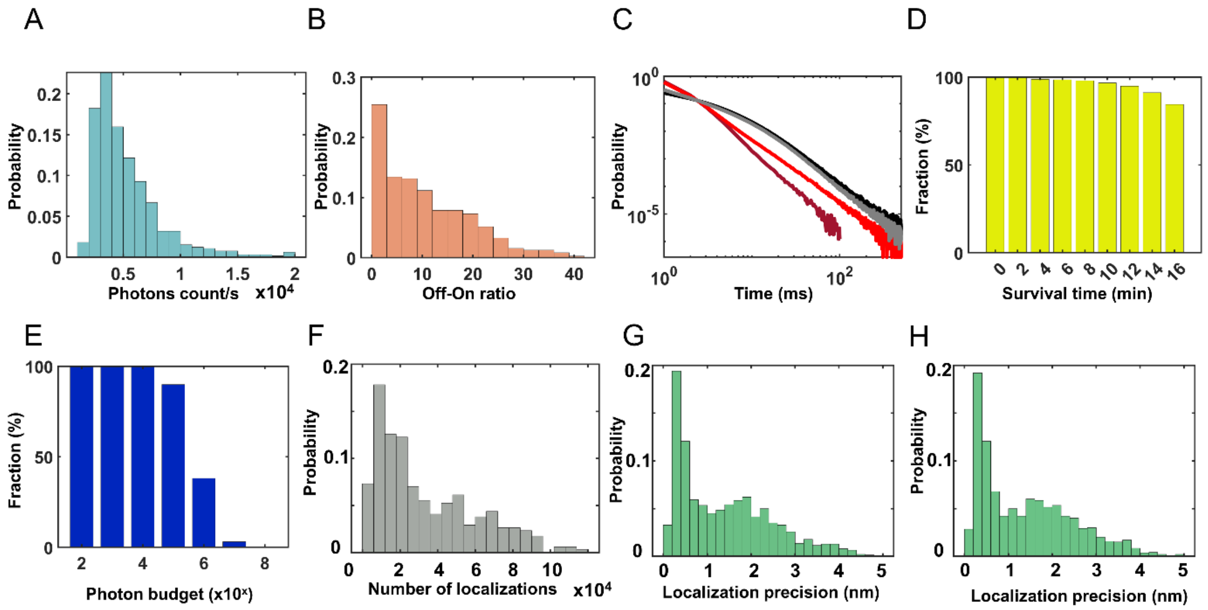

Fig. S5. Photo-physics characterization of mPIEZO1 protein in vitrified cell membrane. The photo-physics characterization (A-E) is an outcome of an average of 8 fields of view (FOV), N= 660 molecules (all detected molecules, not filtered). **(A)** Overall histogram of photon counts/s. **(B)** Photo-blinking performance of the fluorophores in the sample. We observe a broad range of off-on ratios, likely affected by the local environment. Importantly, the off-times are longer than the on-times, facilitating efficient super-resolution imaging. **(C)** Probability distributions of the on and off times are presented on a log-log plot. The on-times distribution from a single FOV is shown in dark red, and from all FOVs in light red. The off-times distribution from a single FOV is shown in black, and from all FOVs in gray. **(D)** Fraction of molecules that survive bleaching up to a given point of time. **(E)** Photon budget per molecule, namely display the fraction of molecules that at least 10^x photons were collected along the experiment, where x is indicated on the x-axis of the bar histogram. **(F)** Average number of localizations registered per molecules from the two cameras. **(G)** Localization precision in the x axis. **(H)** Localization precision in the y-axis.

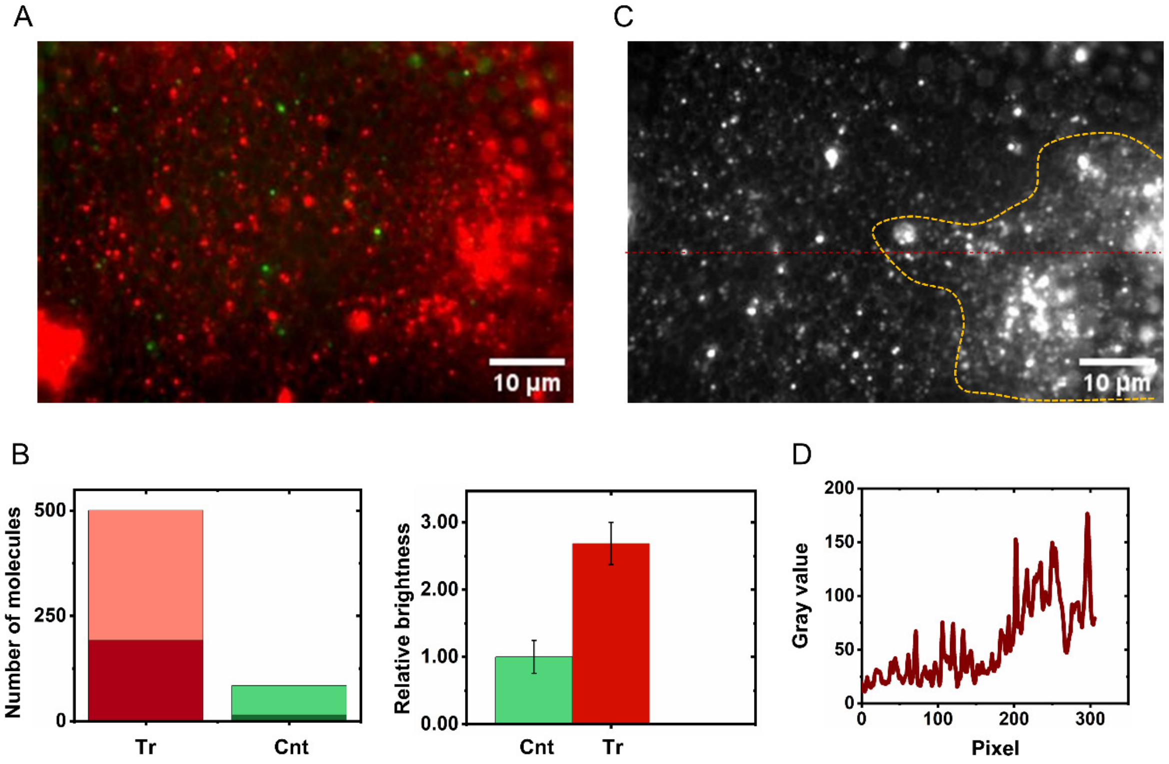

Fig. S6. Single-molecule imaging of unroofed COS7 cells expressing mPIEZO1 protein. (A) Non-transfected labelled unroofed cell were studied to characterize and quantify the number of single molecules observed per field of view (FOV) compared to transfected labelled cells. Here, the non-transfected labeled cells were prepared exactly as described in the routine outlined in the Methods section of the main manuscript and Fig. S2, but without the plasmid expressing mPIEZO1. 5000 frames were summed for each condition and merged together for comparison. Non-transfected cells are shown in green, while the transfected one is displayed in red. It is clear that the number of PSF in the green channel is much lower and dimmer than the ones in the red channel, indicating low background molecules. The green bright spots are likely auto-fluorescent molecules in the cell (see movies S4-5). **(B)** Total number of molecules detected in each FOV, as well as the brightness of the detected molecules. The number of molecules before filtrations is shown in light red for the transfected labelled cells (Tr) and in dark red after filtration, compared to the non-transfected cells in green (Cnt). The brightness of the transfected labelled cell is much higher (~ x2.5) than that of transfected labelled cells (same labelling code), which reduces the potential of background artefacts. **(C)** Same FOV presented in (A), but for the transfected labelled cells, where we indicate the presence of high background area (orange dashed line). **(D)** A cross section profile (red dashed line) shows the relative brightness across the grid.

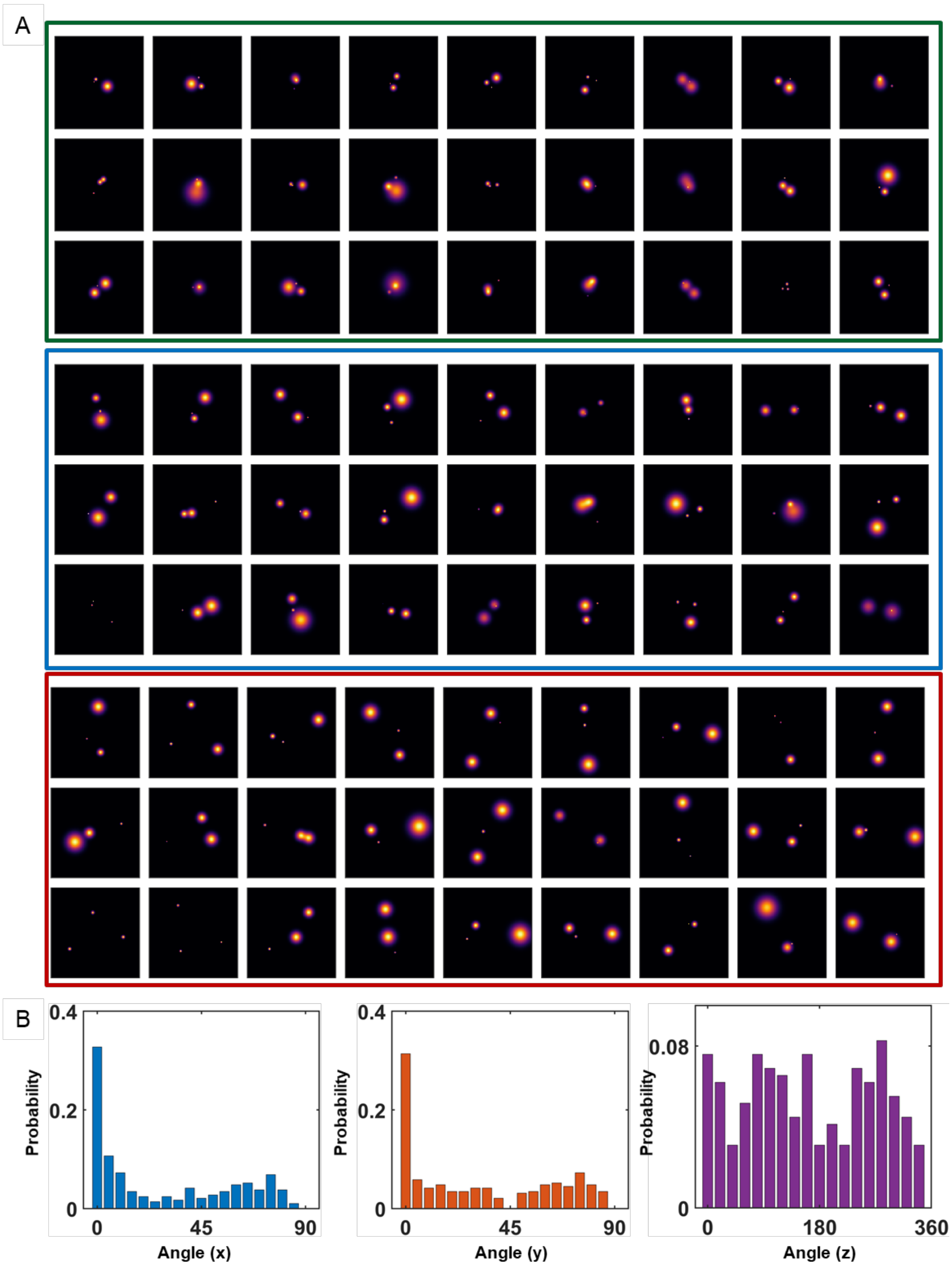

Fig. S7. 2D resolved images of three fluorophores obtained from mPIEZO1 protein sample. (A) Examples of the resolved 2D maps as obtained from polarization trace fitted best with a three-state model. Particles were filtered based on localization precision better than 2 nm and classified to the three different configurations as explained in Fig. S11, (green = class i (9 nm), blue = class ii (19 nm), and red= class ii (34 nm)). The image size is 300 × 300 pixel at 0.2 nm/pixel. We note that two localizations may appear as a single spot for a given particle orientation in the image plane if they overlap within the measurement precision. **(B)** Particle orientation in 3D as obtained from the simulated annealing algorithm (see method section in the main text). The x-axis (pitch) and y-axis (roll) represent the out-of-plane orientations, while the z-axis (yaw) represents the in-plane orientation.

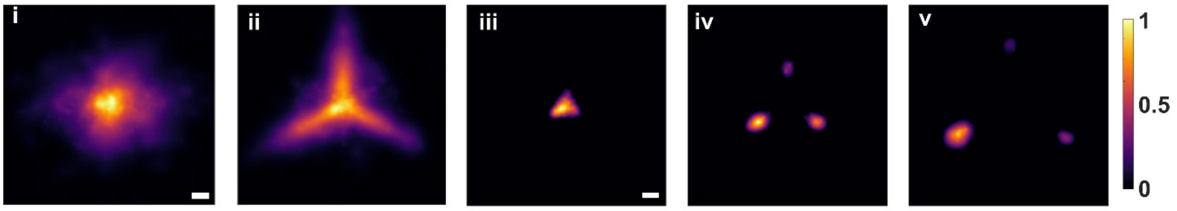

Fig. S8. mPIEZO1 conformational states. To gain insight about the structural conformations of the mPIEZO1 protein, we summed all 2D resolved images (i), and rotated them to align on top of each other (ii). Then, we plotted the projections selected from the three peaks resolved in the pairwise distance histogram. Images show the projections of molecules with maximum distance in the range 7-12 nm (iii), 17-25 nm (iv) and 29-37 nm (v). These plots indicate symmetric conformations match the selected peaks of 9, 19 and 34 nm. The images are composed of 300 x 300 pixels with a resolution of 0.2nm/pixel. Scale bar is 5 nm.

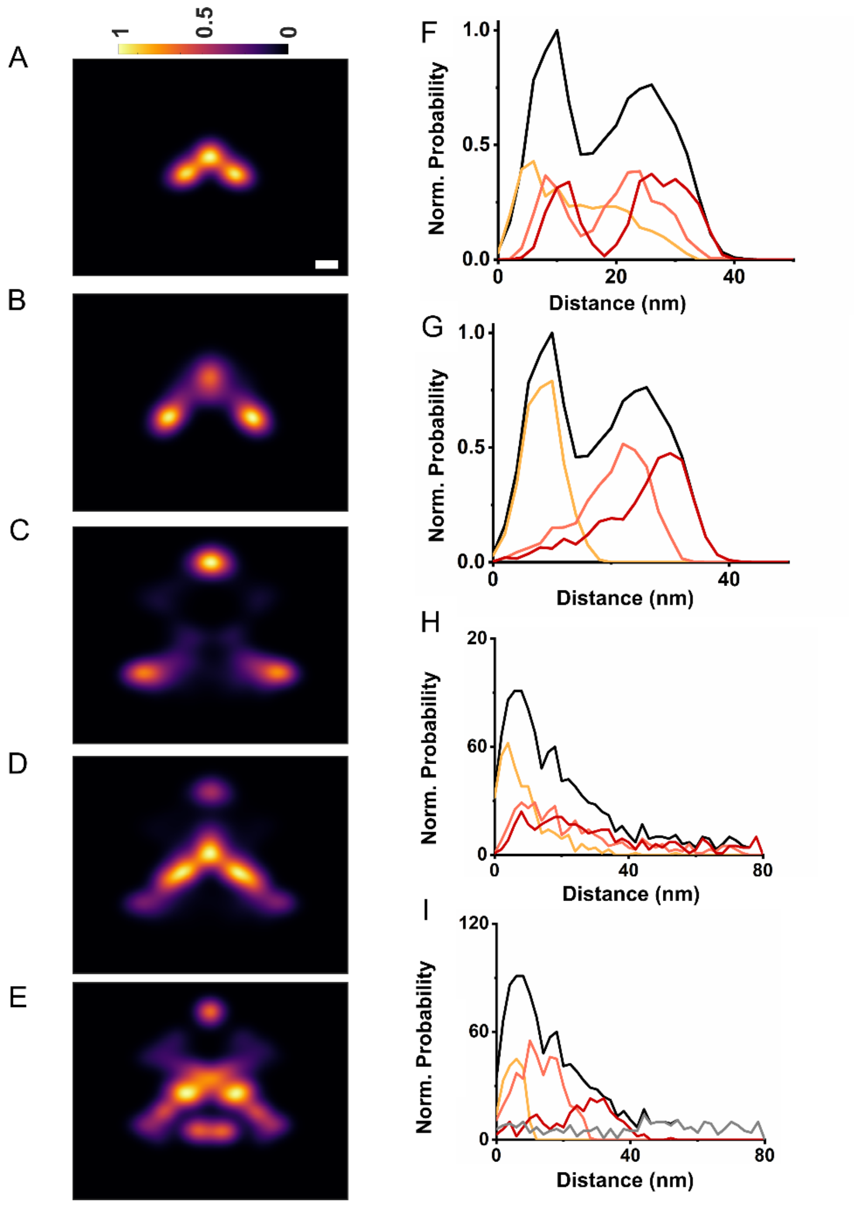

Fig. S9. Structural model assessment based on 2D resolved image and distance histograms. In general, the main modes in the distance histogram indicates the dominant side length in the sample. These multiple side-lengths might arise from the same molecule in case of asymmetric structure, or from the presence of multiple symmetric structures, where each structure shares a distinct single side-length. To differentiate between the two possibilities, one can align all the 2D projection in the sample on top of each other and identify a pattern based on the location density. **(A-C)** Sum of aligned 2D maps of single symmetric conformations with a single side length of 10, 23 and 32 nm, respectively, generated based on simulation with a localization precision of 2 nm. Clearly, each image provides a feature of the structure under study, which is not possible to get from individual unaligned single projections. For example, by taking the coordinates of the maximum density in each map, one can calculate the side length and learn about the symmetry of the structure. In the case of (A-C), it is evident that the structures are equilateral triangles. **(D)** The Y-like shape pattern results from the sum of the aligned 2D projection a because all three projection are present in the sample. **(E)** If we consider an asymmetric triangle shape, sharing a combination of the mentioned side lengths, one would obtain an X-like shape structure. In addition, one can separate the two models based on the pairwise distance histogram. Here, one can sort the distance in each molecule from low to high, and then plot the

histogram of the low, mid, and high distance. **(F)** Simulation of combined multiple symmetric structures (10, 23 and 32 nm), shows that there is no clear separation of the distance distributions (low = orange curve, mid = light red curve and high = dark red curve). **(G)** In the case of simulated asymmetric structure with the same side lengths, the distance histograms are clearly separated. **(H)** We performed this exercise on our experimental data, and we can clearly see that not all the distance distributions are separated, confirming our hypothesis of multiple symmetric conformations. **(I)** Upon classification of our experimental data based on template matching, we plotted the distance histogram for each class separately. As one can notice, we were able to segment the overall distance histogram correctly, indicating proper classification and confirming our result of the presence of multiple symmetric conformation, sharing a distinct side length of 9, 19 and 34 nm. The grey curve in represent the 94 unclassified particles that yield a large side length of more than 43 nm (see Fig. S11). Scale bar in (A-E) is 5 nm.

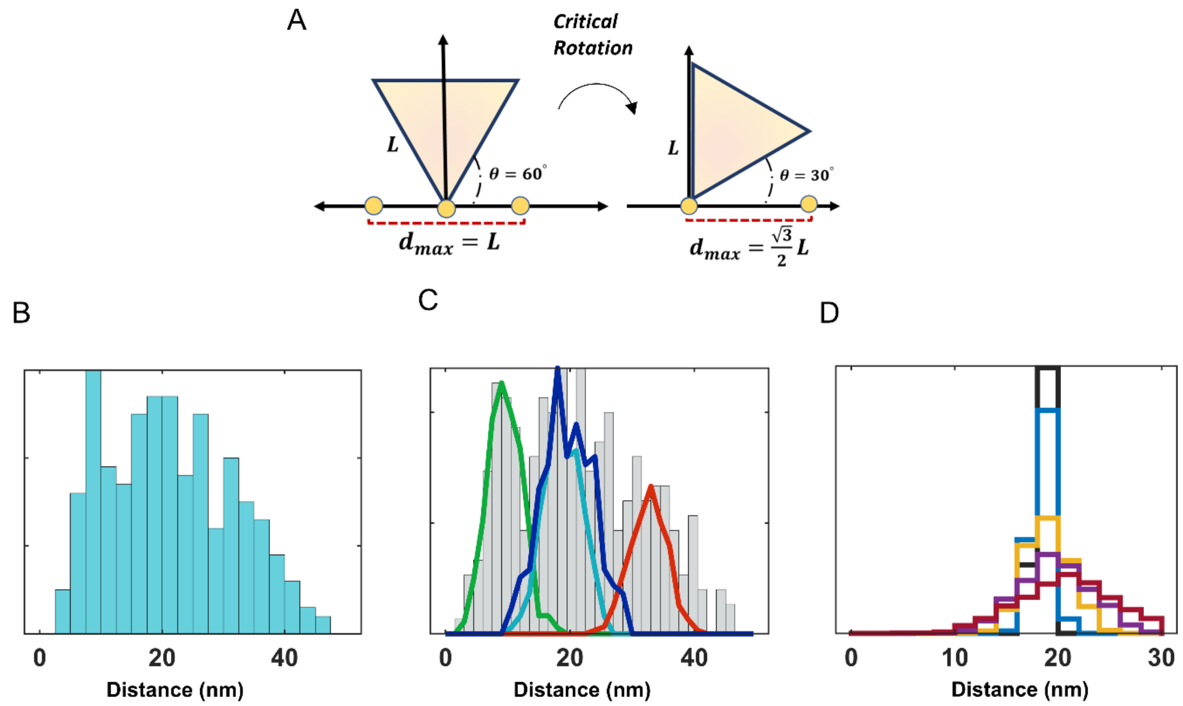

Fig. S10. Maximum side length validation. (A) Distance projection on the 2D plane from an equilateral triangle with side length L. The scheme shows the variation of the side lengths as a function of rotation. In most cases, at least one of the side lengths maintains an absolute distance in the 2D plane, with a minimum projection obtained at a 30-degree rotation (critical rotation), which yields $\frac{\sqrt{3}}{2}L$. **(B)** Maximum side-length histogram as in Fig. 3I (main text) but with 2.5 binning size. The three peaks are at the same position as shown previously (Fig. 3I, main text). **(C)** Validation of the experimental maximum side-length against simulation. The maximum side-length histogram with 1.5 binning size as in Fig. 3I (main text) shown in gray. Green curve plots the maximum side length histogram of simulated equilateral triangle with 9 nm side length and 1.4 nm localization precision. Light and dark blue curves are the maximum side-length histograms of simulated equilateral triangle with 19 nm side length and 1.4 nm and 1.7 nm localization precision, respectively. Red curve is the maximum side-length histogram of simulated equilateral triangle with 34 nm side length and 1.4 nm localization precision. The data show that the broadening in our experimental maximum side-length is localization precision limited. **(D)** Broadening of a maximum side-length histogram of simulated equilateral triangle with 19 nm side length as a function of localization precision 0 nm (black line), 0.5 nm (blue line), 1.4 nm (orange line), 2 nm (purple line) and 3 nm (dark red line).

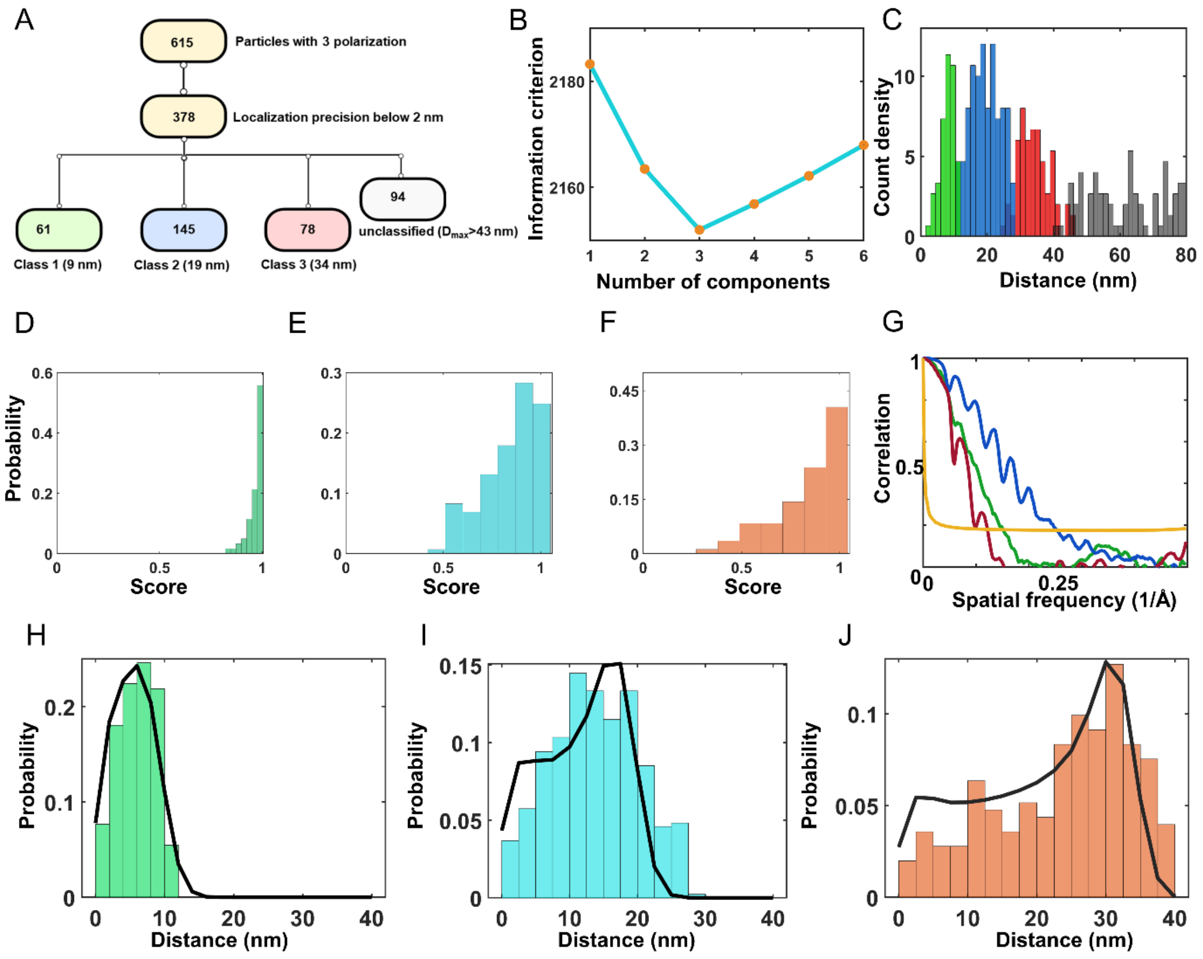

Fig. S11. 2D Single-particle classification and 3D reconstruction. (A) Number of identified molecules along the classification pipeline. Overall, we obtain a high yield > 45%. **(B)** The Akaike information criterion obtained from Gaussian mixture model fitted with different number of components. The minimum value indicates 3 components as the best to describe the data. **(C)** Maximum distance histogram, which is segmented based on the particle calcification. Green is class i, blue is class ii, red is class iii and black are the unclassified particles. **(D-F)** Score metrics for particle classification for each class following the same color code indicated previously. The color code is the same for (C). **(G)** Fourier shell correlation (FSC) obtained for the 3D reconstituted volumes of class i-iii (green, blue, red curve, respectively) yielding 4-8 Å resolution based on half-bit criteria (*45*). **(H-J)** Distance histograms of each class separately, fitted with a model that takes the orientation and localization precision into account. The fit yields a side length of 8.8, 18.6 and 34.5 nm for each class, respectively.

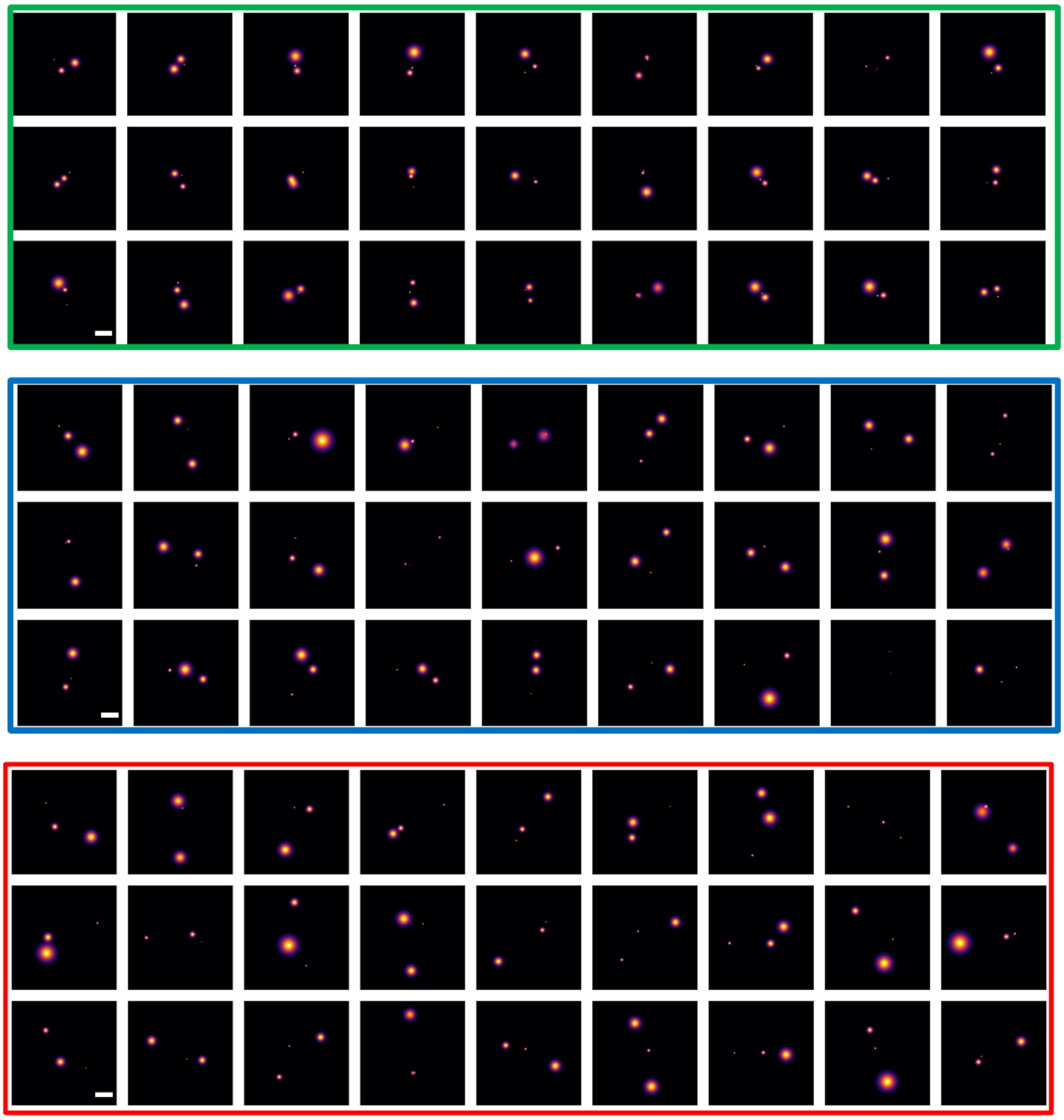

Fig. S12. 2D resolved images of three fluorophores obtained from mPIEZO1-S2472E mutant. Examples of the resolved 2D maps as obtained from polarization trace fitted best with a three-state model. Particles were filtered based on localization precision better than 2 nm and classified to the three different configurations as explained in Fig. S11, (green = class i (9 nm), blue = class ii (24 nm), and red= class ii (34 nm)). The image size is 300 × 300 pixel at 0.2 nm/pixel. We note that two localizations may appear as a single spot for a given particle orientation in the image plane if they overlap within the measurement precision. Scale bar is 10 nm.

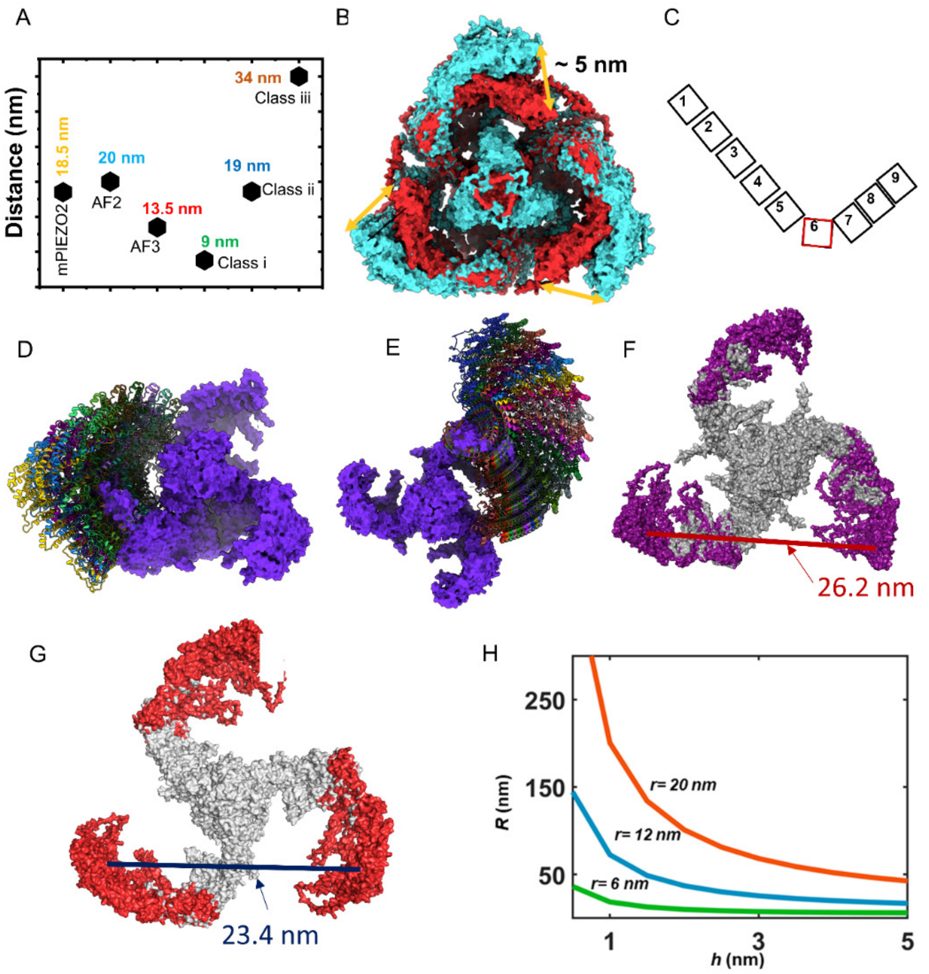

Fig. S13. Predicted mPIEZO1 blade conformational states. (A) Summary of the blade-blade distance with respect to amino acid 103 as obtained for different PIEZO protein models, mPIEZO1, mPIEZO2 obtained from (PDB: 6KG7), AlphaFold 2 (AF2) predication (E2JF22), AlphaFold 3 (AF3) predication, and class i-iii are our experimental data. **(B)** Full-length structural model of AF2 protein (E2JF22, cyan color) and AF3 (red color). The blade structure of the model obtained from AF3 bent more toward the centre of the protein by additional ~ 5 nm, yields 20 nm for AF2 and 13.5 nm for AF3. **(C)** Blade structure indicates the position of the elbow (*3*), as well as the pivot point used for rigid body rotation (see Methods in the main text). **(D-E)** To test whether any structural state of the blades can explain the 9 nm and 34 nm distance, we used a pivot point on the "elbow" of the blades as mentioned in (C). The shorter distance of 9 nm could be explained by a severe bend of the blade toward the center as predicted by AF3 model (A), and simultaneously by moving the blades upward as shown in panel (D), which results in shorter end-end distance that matches our experimentally measured distance (see Method in the main text). The large distance of 34 nm can be explained by unbending the blade structure using the chimera structure of the fully flat PIEZO model (PDB: 7WLU) and AF2 prediction as indicated in panel (F). We first rotated the blade as a rigid body in the plane. A ~50 degree in-plane rotation yields a straighter blade conformation matches experimentally-measured side length very well. **(F)** To generate the fully flat mPIEZO1 structure, we used the Cryo-EM structure (PDB: 7WLU)(*8*), shown in gray, and extended the unresolved part of the blade domain using AF2 model (purple) with PyMOL software (see Methods section). The inter-blade distance in this case yields ~26 nm. **(G)** To generate the partially flat mPIEZO1 structure, we used the Cryo-EM structure (PDB: 8IXO)(*18*), shown in gray, and extended the unresolved part of the blade domain using AF2 model (red) with PyMOL software (see Methods section). The inter-blade distance in this case yields ~23 nm. **(H)** Calculated radius of curvature (*R*) as a function of the estimate dome height (*h*) (see Fig. 1D) for each class, using the equation in the main text.

Table S1: Comparison of different PIEZO parameters, which to the best of our knowledge, have been used for describing blade conformations measured with various methods. For the reader's convenience, we also mark some of the key parameters in the figure below the table (Fig. S14). All the data are relevant to mPIEZO1, except where indicated.

parameter<br>Ref	year	Method	Environment	Conformation	Inter-blade distance (d), (nm)	In-plane radius (r_{label}), (nm)	In-plane radius ($r_{exterior}$), (nm)	Dome height (h), (nm)	Radius of curvature (R), (nm)
(*3*)	2017	Cryo-EM	liposome	Curved state	-	-	**~ 10**	-	**10**
(7)	2019	AFM	SLB	Curved state (type1)	-	-	**11**	**5**	-
				Flat state (type 1)	-	-	**17**	**assumed ~ 0**	-
				Flat state (type 2)	-	-	**~ 22**	**assumed ~ 0**	-
(*63*)	2019	Cryo-EM	detergent	Curved (PIEZO2)	**~ 19**	**12**	**12**	**9**	**~ 12**
(*9*)	2022	Theoretical model	SLB	Rest state	-	-	-	-	**~ 42**
(*17*)	2022	Light microscopy (STED), and electron microscopy	unroofed red blood cell	-	**~ 25**	**~ 14**	-	-	-
(*8*)	2022	Cryo-EM	liposome	Curved	-	-	**10**	**10**	**10**
				Flat	-	-	**14**	**~ 0**	**∞**
(*16*)	2023	Light microscopy (MINFLUX)	fixed HEK293 cells	Rest state	**25**	**~ 14**	-	-	-
			detergent	Compact	**17**	**~ 10**	-	-	-
			fixed HEK293 cells treated with GsMTx-4	Compact	**20**	**~ 12**	-	-	-
			fixed HEK293 cells – Hypotonic condition	Expanded	**34**	**~ 20**	-	-	-
(*18*)	2024	Cryo-EM	detergent	Curved	-	-	**10**	**5**	**12**
				Intermediate	-	-	**12**	**2.5**	**32**
				Flat	-	-	**12**	**1**	**117**
-	-	AF2	Prediction	Curved	**20**	**12**	**14**	-	-
-	-	AF3	Prediction	Curved	**~13**	**8**	**12**	-	-
This work	2025	Light microscopy (spCryo-LM)	Unroofed COS7 cells	Curved	**9**	**6**	**10**	**~ 5**	**6**
				Rest/intermediate	**19**	**12**	**~14**	**0-5**	**26-35**
				Mutant intermediate	**24**	**~15**	**~15**	**0-5**	**35-47**
				Flat	**34**	**~20**	**~20**	**0**	**∞**

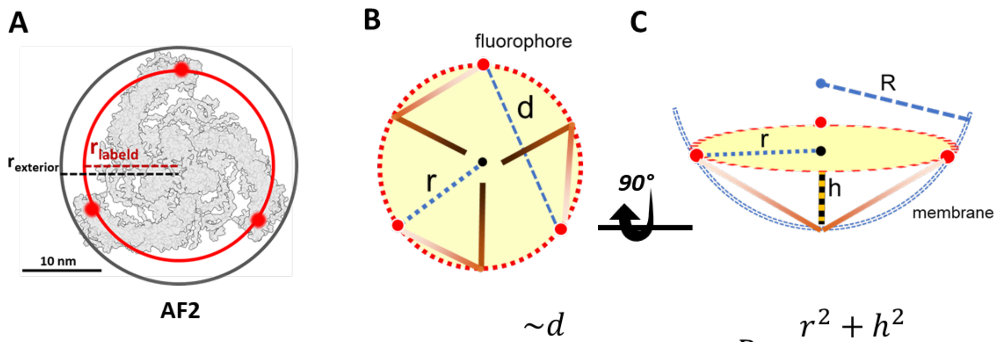

$$r_{label} = \frac{\sim d}{\sqrt{3}}$$

$$R = \frac{r^2 + h^2}{2h}$$

Fig. S14. (A) Illustrates the definition of the inter-plane radius (*r*), which is determined by either by the exterior or the position of the fluorophores. **(B)** Outlines the calculation of the inter-plane radius based on the inter-blade distance (*d*) measured from the fluorophore positions. Notably, we added 1.5 nm to the distance d to account for the edge of the blade (see main text for details). **(C)** Describes the calculation of the radius of curvature. It is important to note that each of the listed methods can directly capture some of these parameters, but not all of them.

Legends for Movies S1 to S9

Movie S1: Step-by-step transfer of vitrified samples at high vacuum and cryogenic temperature. The video shows the entire process of transferring the vitrified sample into the microscope. The process can also be executed in reverse order for grid retrieval for subsequent analyses and correlative imaging. The video has been sped up 4x to reduce its size.

Movie S2: Imaging vitrified aqueous solution of ATTO647N. Fluorescence images of a vitrified aqueous solution of ATTO647N recorded on a carbon mesh TEM grid. The video was recorded on one of the cameras after polarization splitting at 70 Hz.

Movie S3: Imaging mPIEZO1 in its near-native cell membrane. Fluorescence images of vitrified samples of mPIEZO1 expressed in the COS7 cell membrane were recorded on one of the cameras after polarization splitting at 70 Hz. The video shows one scenario of sparse PSFs with good off-on ratio.

Movie S4: Imaging mPIEZO1 in its near-native cell membrane. Fluorescence images of vitrified samples of mPIEZO1 expressed in the COS7 cell membrane were recorded on one of the cameras after polarization splitting at 70 Hz. The video shows another scenario of sparse PSFs.

Movie S5: Control measurement of non-transfected labelled unroofed COS7 cells to assess the background at cryogenic temperatures. Fluorescence images of vitrified samples of unroofed COS7 cell membranes were recorded on one of the cameras after polarization splitting at 70 Hz. The video shows a significantly lower number of molecules compared to transfected, labeled cells. Additionally, the fluorescent signal is considerably weaker than that of transfected, labeled cells (see Fig. S6).

Movie S6: 3D reconstruction of mPIEZO1 class i conformation.

Movie S7: 3D reconstruction of mPIEZO1 class ii conformation.

Movie S8: 3D reconstruction of mPIEZO1 class iii conformation.

Movie S9: 3D structural models of mPIEZO1 blade conformations. The video shows the structural conformations of the blade domain that match our experimentally resolved configurations (classes i–iii), as described in Fig. 4 and 6, Methods section, and Fig. S13.

REFERENCES AND NOTES

1. B. Xiao, Mechanisms of mechanotransduction and physiological roles of PIEZO channels. *Nat. Rev. Mol. Cell Biol.* **25**, 886–903 (2024).

2. B. Coste, J. Mathur, M. Schmidt, T. J. Earley, S. Ranade, M. J. Petrus, A. E. Dubin, A. Patapoutian, Piezo1 and Piezo2 are essential components of distinct mechanically activated cation channels. *Science* **330**, 55–60 (2010).

3. Y. R. Guo, R. MacKinnon, Structure-based membrane dome mechanism for Piezo mechanosensitivity. *Elife* **6**, e33660 (2017).

4. J. Ge, W. Li, Q. Zhao, N. Li, M. Chen, P. Zhi, R. Li, N. Gao, B. Xiao, M. Yang, Architecture of the mammalian mechanosensitive Piezo1 channel. *Nature* **527**, 64–69 (2015).

5. K. Saotome, S. E. Murthy, J. M. Kefauver, T. Whitwam, A. Patapoutian, A. B. Ward, Structure of the mechanically activated ion channel Piezo1. *Nature* **554**, 481–486 (2018).

6. Q. Zhao, H. Zhou, S. Chi, Y. Wang, J. Wang, J. Geng, K. Wu, W. Liu, T. Zhang, M.-Q. Dong, J. Wang, X. Li, B. Xiao, Structure and mechanogating mechanism of the Piezo1 channel. *Nature* **554**, 487–492 (2018).

7. Y.-C. Lin, Y. R. Guo, A. Miyagi, J. Levring, R. Mackinnon, S. Scheuring, Force-induced conformational changes in PIEZO1. *Nature* **573**, 230–234 (2019).

8. X. Yang, C. Lin, X. Chen, S. Li, X. Li, B. Xiao, Structure deformation and curvature sensing of PIEZO1 in lipid membranes. *Nature* **604**, 377–383 (2022).

9. C. A. Haselwandter, Y. R. Guo, Z. Fu, R. MacKinnon, Elastic properties and shape of the Piezo dome underlying its mechanosensory function. *Proc. Natl. Acad. Sci. U.S.A.* **119**, e2208034119 (2022).

10. C. A. Haselwandter, R. MacKinnon, Piezo's membrane footprint and its contribution to mechanosensitivity. *Elife* **7**, e41968 (2018).

11. M. Young, A. H. Lewis, J. Grandl, Physics of mechanotransduction by Piezo ion channels. *J. Gen. Physiol.* **154**, e202113044 (2022).

12. C. A. Haselwandter, Y. R. Guo, Z. Fu, R. MacKinnon, Quantitative prediction and measurement of Piezo's membrane footprint. *Proc. Natl. Acad. Sci.* **119**, e2208027119 (2022).

13. J. M. Kefauver, A. B. Ward, A. Patapoutian, Discoveries in structure and physiology of mechanically activated ion channels. *Nature* **587**, 567–576 (2020).

14. L. O. Romero, A. E. Massey, A. D. Mata-Daboin, F. J. Sierra-Valdez, S. C. Chauhan, J. F. Cordero-Morales, V. Vásquez, Dietary fatty acids fine-tune Piezo1 mechanical response. *Nat. Commun.* **10**, 1200 (2019).

15. P. Ridone, E. Pandzic, M. Vassalli, C. D. Cox, A. Macmillan, P. A. Gottlieb, B. Martinac, Disruption of membrane cholesterol organization impairs the activity of PIEZO1 channel clusters. *J. Gen. Physiol.* **152**, e201912515 (2020).

16. E. M. Mulhall, A. Gharpure, R. M. Lee, A. E. Dubin, J. S. Aaron, K. L. Marshall, K. R. Spencer, M. A. Reiche, S. C. Henderson, T. L. Chew, A. Patapoutian, Direct observation of the conformational states of PIEZO1. *Nature* **620**, 1117–1125 (2023).

17. G. Vaisey, P. Banerjee, A. J. North, C. A. Haselwandter, R. MacKinnon, Piezo1 as a force-through-membrane sensor in red blood cells. *Elife* **11**, e82621 (2022).

18. S. Liu, X. Yang, X. Chen, X. Zhang, J. Jiang, J. Yuan, W. Liu, L. Wang, H. Zhou, K. Wu, B. Tian, X. Li, B. Xiao, An intermediate open structure reveals the gating transition of the mechanically activated PIEZO1 channel. *Neuron* **113**, 1–15 (2024).

19. S. C. M. Reinhardt, L. A. Masullo, I. Baudrexel, P. R. Steen, R. Kowalewski, A. S. Eklund, S. Strauss, E. M. Unterauer, T. Schlichthaerle, M. T. Strauss, C. Klein, R. Jungmann, Ångström-resolution fluorescence microscopy. *Nature* **617**, 711–716 (2023).

20. S. J. Sahl, J. Matthias, K. Inamdar, M. Weber, T. A. Khan, C. Brüser, S. Jakobs, S. Becker, C. Griesinger, J. Broichhagen, S. W. Hell, Direct optical measurement of intramolecular distances with angstrom precision. *Science* **386**, 180–187 (2024).

21. T. Ichikawa, D. Wang, K. Miyazawa, K. Miyata, M. Oshima, T. Fukuma, Chemical fixation creates nanoscale clusters on the cell surface by aggregating membrane proteins. *Commun. Biol.* **5**, 487 (2022).

22. P. M. Pereira, D. Albrecht, S. Culley, C. Jacobs, M. Marsh, J. Mercer, R. Henriques, Fix your membrane receptor imaging: Actin cytoskeleton and CD4 membrane organization disruption by chemical fixation. *Front. Immunol.* **10**, 675 (2019).

23. N. Korogod, C. C. H. Petersen, G. W. Knott, Ultrastructural analysis of adult mouse neocortex comparing aldehyde perfusion with cryo fixation. *Elife* **4**, e05793 (2015).

24. J. Dubochet, M. Adrian, J.-J. Chang, J.-C. Homo, J. Lepault, A. W. McDowall, P. Schultz, Cryo-electron microscopy of vitrified specimens. *Q. Rev. Biophys.* **21**, 129–228 (1988).

25. I. Hurbain, M. Sachse, The future is cold: Cryo-preparation methods for transmission electron microscopy of cells. *Biol. Cell* **103**, 405–420 (2011).

26. J. Dubochet, The physics of rapid cooling and its implications for cryoimmobilization of cells. *Methods Cell Biol.* **79**, 7–21 (2007).

27. D. P. Hoffman, G. Shtengel, C. S. Xu, K. R. Campbell, M. Freeman, L. Wang, D. E. Milkie, H. A. Pasolli, N. Iyer, J. A. Bogovic, D. R. Stabley, A. Shirinifard, S. Pang, D. Peale, K. Schaefer, W. Pomp, C. L. Chang, J. Lippincott-Schwartz, T. Kirchhausen, D. J. Solecki, E. Betzig, H. F. Hess, Correlative three-dimensional super-resolution and block-face electron microscopy of whole vitreously frozen cells. *Science* **367**, eaaz5357 (2020).

28. P. D. Dahlberg, S. Saurabh, A. M. Sartor, J. Wang, P. G. Mitchell, W. Chiu, L. Shapiro, W. E. Moerner, Cryogenic single-molecule fluorescence annotations for electron tomography reveal in situ organization of key proteins in Caulobacter. *Proc. Natl. Acad. Sci. U.S.A.* **117**, 13937–13944 (2020).

29. M. W. Tuijtel, A. J. Koster, S. Jakobs, F. G. A. Faas, T. H. Sharp, Correlative cryo super-resolution light and electron microscopy on mammalian cells using fluorescent proteins. *Sci. Rep.* **9**, 1369 (2019).

30. P. D. Dahlberg, W. E. Moerner, Cryogenic super-resolution fluorescence and electron microscopy correlated at the nanoscale. *Annu. Rev. Phys. Chem.* **72**, 253–278 (2021).

31. H. Mazal, F. F. Wieser, V. Sandoghdar, Insights into protein structure using cryogenic light microscopy. *Biochem. Soc. Trans.* **51**, 2041–2059 (2023).

32. H. Mazal, F.-F. Wieser, D. Bollschweiler, V. Sandoghdar, Cryogenic light microscopy of vitrified samples with Ångstrom precision. bioRxiv 2025.05.27.656160 [Preprint] (2025). https://doi.org/10.1101/2025.05.27.656160.

33. S. Tacke, V. Krzyzanek, H. Nusse, R. A. Wepf, J. Klingauf, R. Reichelt, A versatile high-vacuum cryo-transfer system for cryo-microscopy and analytics. *Biophys. J.* **110**, 758–765 (2016).

34. G. McMullan, K. R. Vinothkumar, R. Henderson, Thon rings from amorphous ice and implications of beam-induced Brownian motion in single particle electron cryo-microscopy. *Ultramicroscopy* **158**, 26–32 (2015).

35. N. Biyani, R. D. Righetto, R. McLeod, D. Caujolle-Bert, D. Castano-Diez, K. N. Goldie, H. Stahlberg, Focus: The interface between data collection and data processing in cryo-EM. *J. Struct. Biol.* **198**, 124–133 (2017).

36. S. Weisenburger, D. Boening, B. Schomburg, K. Giller, S. Becker, C. Griesinger, V. Sandoghdar, Cryogenic optical localization provides 3D protein structure data with Angstrom resolution. *Nat. Methods* **14**, 141–144 (2017).

37. D. Böning, F.-F. Wieser, V. Sandoghdar, Polarization-encoded colocalization microscopy at cryogenic temperatures. *ACS Photonics* **8**, 194–201 (2021).

38. H. Mazal, F. F. Wieser, V. Sandoghdar, Deciphering a hexameric protein complex with Angstrom optical resolution. *Elife* **11**, e76308 (2022).

39. Y. Zhang, H. Mazal, V. S. Mandala, G. Perez-Mitta, V. Sondoghdar, C. A. Haselwandter, R. MacKinnon, Higher-order transient membrane protein structures. *Proc. Natl. Acad. Sci. U.S.A.* **122**, e2421275121 (2025).

40. C. F. Peitsch, S. Beckmann, B. Zuber, iMEM: Isolation of plasma membrane for cryoelectron microscopy. *Structure* **24**, 2198–2206 (2016).

41. M. Clarke, G. Schatten, D. Mazia, J. A. Spudich, Visualization of actin fibers associated with the cell membrane in amoebae of Dictyostelium discoideum. *Proc. Natl. Acad. Sci. U.S.A.* **72**, 1758–1762 (1975).

42. N. Morone, E. Usukura, A. Narita, J. Usukura, Improved unroofing protocols for cryo-electron microscopy, atomic force microscopy and freeze-etching electron microscopy and the associated mechanisms. *Microscopy* **69**, 350–359 (2020).

43. W. W. Sun, D. J. Michalak, K. A. Sochacki, P. Kunamaneni, M. A. Alfonzo-Méndez, A. M. Arnold, M.-P. Strub, J. E. Hinshaw, J. W. Taraska, Cryo-electron tomography pipeline for plasma membranes. *Nat. Commun.* **16**, 855 (2025).

44. N. C. Dvornek, F. J. Sigworth, H. D. Tagare, SubspaceEM: A fast maximum-a-posteriori algorithm for cryo-EM single particle reconstruction. *J. Struct. Biol.* **190**, 200–214 (2015).

45. M. Van Heel, M. Schatz, Fourier shell correlation threshold criteria. *J. Struct. Biol.* **151**, 250–262 (2005).

46. J. Jumper, R. Evans, A. Pritzel, T. Green, M. Figurnov, O. Ronneberger, K. Tunyasuvunakool, R. Bates, A. Žídek, A. Potapenko, A. Bridgland, C. Meyer, S. A. A. Kohl, A. J. Ballard, A. Cowie, B. Romera-Paredes, S. Nikolov, R. Jain, J. Adler, T. Back, S. Petersen, D. Reiman, E. Clancy, M. Zielinski, M. Steinegger, M. Pacholska, T. Berghammer, S. Bodenstein, D. Silver, O. Vinyals, A. W. Senior, K. Kavukcuoglu, P. Kohli, D. Hassabis, Highly accurate protein structure prediction with AlphaFold. *Nature* **596**, 583–589 (2021).

47. J. Abramson, J. Adler, J. Dunger, R. Evans, T. Green, A. Pritzel, O. Ronneberger, L. Willmore, A. J. Ballard, J. Bambrick, S. W. Bodenstein, D. A. Evans, C.-C. Hung, M. O'Neill, D. Reiman, K. Tunyasuvunakool, Z. Wu, A. Žemgulytė, E. Arvaniti, C. Beattie, O. Bertolli, A. Bridgland, A. Cherepanov, M. Congreve, A. I. Cowen-Rivers, A. Cowie, M. Figurnov, F. B. Fuchs, H. Gladman, R. Jain, Y. A. Khan, C. M. R. Low, K. Perlin, A. Potapenko, P. Savy, S. Singh, A. Stecula, A. Thillaisundaram, C. Tong, S. Yakneen, E. D. Zhong, M. Zielinski, A. Žídek, V. Bapst, P. Kohli, M. Jaderberg, D. Hassabis, J. M. Jumper, Accurate structure prediction of biomolecular interactions with AlphaFold 3. *Nature* **630**, 493–500 (2024).

48. I. G. Denisov, S. G. Sligar, Nanodiscs for structural and functional studies of membrane proteins. *Nat. Struct. Mol. Biol.* **23**, 481–486 (2016).

49. A. Helenius, K. Simons, Solubilization of membranes by detergents. *Biochim. Biophys. Acta* **415**, 29–79 (1975).

50. T. J. Knowles, R. Finka, C. Smith, Y.-P. Lin, T. Dafforn, M. Overduin, Membrane proteins solubilized intact in lipid containing nanoparticles bounded by styrene maleic acid copolymer. *J. Am. Chem. Soc.* **131**, 7484–7485 (2009).

51. J. M. Voss, O. F. Harder, P. K. Olshin, M. Drabbels, U. J. Lorenz, Rapid melting and revitrification as an approach to microsecond time-resolved cryo-electron microscopy. *Chem. Phys. Lett.* **778**, 138812 (2021).

52. J. Michaelis, C. Hettich, J. Mlynek, V. Sandoghdar, Optical microscopy using a single-molecule light source. *Nature* **405**, 325–328 (2000).

53. M. Roseman, P. Grütter, Cryogenic magnetic force microscope. *Rev. Sci. Instrum.* **71**, 3782–3787 (2000).

54. L. Qin, J. Zhang, J. Sun, D. M. Czajkowsky, Z. Shao, Development of a low-noise cryogenic atomic force microscope for high resolution imaging of large biological complexes. *Acta Biochim. Biophys. Sin.* **48**, 859–861 (2016).

55. R. K. Hylton, M. T. Swulius, Challenges and triumphs in cryo-electron tomography. *iScience* **24**, 102959 (2021).

56. M. Turk, W. Baumeister, The promise and the challenges of cryo-electron tomography. *FEBS Lett.* **594**, 3243–3261 (2020).

57. D. N. Mastronarde, Automated electron microscope tomography using robust prediction of specimen movements. *J. Struct. Biol.* **152**, 36–51 (2005).

58. R. Serfling, C. Lorenz, M. Etzel, G. Schicht, T. Böttke, M. Mörl, I. Coin, Designer tRNAs for efficient incorporation of non-canonical amino acids by the pyrrolysine system in mammalian cells. *Nucleic Acids Res.* **46**, 1–10 (2017).

59. J. C. Crocker, D. G. Grier, Methods of digital video microscopy for colloidal studies. *J. Colloid Interface Sci.* **179**, 298–310 (1996).

60. D. S. White, M. P. Goldschen-Ohm, R. H. Goldsmith, B. Chanda, Top-down machine learning approach for high-throughput single-molecule analysis. *Elife* **9**, e53357 (2020).

61. T. D. Goddard, C. C. Huang, E. C. Meng, E. F. Pettersen, G. S. Couch, J. H. Morris, T. E. Ferrin, UCSF ChimeraX: Meeting modern challenges in visualization and analysis. *Protein Sci.* **27**, 14–25 (2018).

62. X. Tao, C. Zhao, R. Mackinnon, Membrane protein isolation and structure determination in cell-derived membrane vesicles. *Proc. Natl. Acad. Sci. U.S.A.* **120**, e2302325120 (2023).

63. L. Wang, H. Zhou, M. Zhang, W. Liu, T. Deng, Q. Zhao, Y. Li, J. Lei, X. Li, B. Xiao, Structure and mechanogating of the mammalian tactile channel PIEZO2. *Nature* **573**, 225–229 (2019).
